# Impact of molar teeth distalization by clear aligners on temporomandibular joint: a three-dimensional study

**DOI:** 10.1186/s40510-023-00474-3

**Published:** 2023-07-17

**Authors:** Barakat Al-Tayar, Majedh A. A. Al-Somairi, Lina H. ALshoaibi, Xiaoli Wang, Junbin Wang, Jiajie Liu, Baher Al-Tayar, Xiaoli An, Qingzong Si

**Affiliations:** 1 Department of Orthodontics, School of Stomatology, Lanzhou University, Lanzhou, 730000 China; 2 Orthodontics Division, Faculty of Medicine and Health Sciences, Taiz University, Taiz, Yemen; 3 Orthodontics Department, School of Stomatology, China Medical University, Shenyang, 110000 China; 4 Graduate Student of Dental Health Science, Faculty of Dental Medicine, Universitas Airlangga, Surabaya, Indonesia; 5 Department of Dentistry, Faculty of Medical Sciences, Aljanad University for Science and Technology, Taiz, Yemen; 6 Department of Oral Medicine, School of Stomatology, Lanzhou University, Lanzhou, 730000 China

**Keywords:** Class II malocclusion, Clear aligners, Molar distalization, Temporomandibular joint, Cone-beam computed tomography, 3D analysis

## Abstract

**Background:**

Maxillary molar distalization is a common technique used in the non-extraction treatment of Angle Class II malocclusion that can effectively correct the molar relationship and create spaces for anterior teeth alignment. However, this approach may also impact the temporomandibular joint (TMJ) due to predictable changes in the posterior vertical dimension. Despite its widespread use, Class II malocclusions correction by molar distalization with clear aligners has not been investigated for their effects on the TMJ. Therefore, this study aimed to analyze the impact of sequential molar distalization using clear aligners on the TMJ.

**Methods:**

Three-dimensional CBCT scans of 23 non-growing patients (7 males, 16 females; mean age 29.8 ± 4.6 years) with skeletal class I or II malocclusion and a bilateral molar class II relationship treated by sequential upper molars distalization with orthodontic clear aligners (Invisalign, Align Technology, San Josè, Ca, USA). A total of 46 joints were examined before and after molar distalization using Anatomage InvivoDental 6.0.3. Linear and angular measurements of the mandibular joint were measured, including joint parameters, inclination, position, and the dimension of the condyle and articular fossa. In addition, 3D volumetric spaces of the joint were analyzed. All data were statistically analyzed by paired T test to determine the differences between the pre-and post-orthodontic procedures.

**Results:**

No statistically significant differences were found in all primary effects resulting from maxillary molars distalization by clear aligners on TMJ components measurements and joint spaces between T0 and T1. Meanwhile, statistically significant differences were observed in the linear position of the upper molars and the molar relationship parameter with at least *P* ≤ 0.05.

**Conclusion:**

Treatment by sequential upper molars distalization with clear aligners does not lead to significant TMJ parameters changes in condyle and fossa spaces, dimensions, and positions.

**Supplementary Information:**

The online version contains supplementary material available at 10.1186/s40510-023-00474-3.

## Background

Malocclusions have become a growing concern in oral public health. According to the World Health Organization, malocclusions are now recognized as the third most prevalent oral health concern, following dental caries and periodontal disorders [1]. In particular, Class II malocclusion is a prevalent disorder that can cause a range of esthetic, psychological, and functional disturbances with varying degrees of severity among the population [2, 3], with a worldwide mean disturbance of 19.56% in permanent dentition [4].

Since extractions treatment has been related to adverse side effects such as facial profile worsening and TMJ problems [5], distalization of the maxillary molars is among the most commonly employed non-extraction treatment strategies for Angle Class II malocclusion. This approach is primarily recommended for subjects with dentoalveolar maxillary protrusion or minor skeletal abnormalities, as they are the primary candidates for this technique [6, 7].

Headgear was the first appliance used for molar distalization and has been the most frequently used appliance to correct anteroposterior discrepancies since the 1950s. However, this appliance requires substantial patient cooperation and is esthetically undesirable [8]. In recent years, various techniques have been designed to reduce or eliminate the reliance on patient compliance, including intra-oral appliances with and without skeletal anchorage. The intra-oral fixed Pendulum appliance was introduced by Dr. James Hilgers [9] in 1992 for maxillary molar distalization. As this appliance is fixed in place, patient compliance becomes less of an issue, and forces are constantly applied. It accompanies different condylar pathway alterations documented as a consequence of upper molar distalization [10, 11].

Clear aligners are orthodontic treatment systems introduced as more aesthetically pleasing and convenient substitutes to conventional fixed appliances. They can address various types of malocclusions, including treating class II malocclusion in adult patients through sequential maxillary molar distalization [12, 13].

The association between dental occlusion and temporomandibular disorders (TMD) remains a controversial issue in dentistry. Thus, Manfredini et al. [14] conducted a literature review to investigate the relationship between the features of dental occlusion and TMDs, ultimately concluding that no clear-cut association exists between them. The role of orthodontic treatment in the onset and evolution of TMD has also been a topic of disagreement among clinicians, as previous literature has suggested that orthodontic treatment can both prevent and cause TMD [15, 16]. Some researchers argue that orthodontic therapy can positively change TMJ remodeling, thereby improving the condyle-glenoid fossa relationship [17]. Conversely, others suggest that orthodontic appliances may alter the balance of the occlusal relationship, potentially causing TMDs [18, 19].

Backward positioning of maxillary arch molars can result in an alteration to the position of the teeth and inter-arch relationship, which can cause repositioning of the mandible and potentially affect the position of the condyle. This, in turn, may disrupt the disc-condylar relationship and induce TMD. In addition, patients undergoing orthodontic treatment to correct malocclusions often experience TMJ adaptive bone remodeling [20, 21]. Therefore, it is crucial to investigate the correlation between orthodontic treatment and its impact on TMJ function to understand TMD's development and progression better. TMD affects a significant portion of the population, with prevalence rates ranging from 5% to 12%, and symptoms often worsen with age, particularly during adolescence [22]. TMD is associated with various clinical signs and symptoms, including pain in the TMJ and jaw muscles, poor mandibular movement, jaw joint locking, and joint sounds [23]. Moreover, its etiology is complicated and multifactorial, including biomechanical, biochemical, and psychological factors [24]. Various factors such as malocclusion, orthodontic treatment, bruxism, trauma, hormone imbalance, stress, depression, and anxiety have been hypothesized as contributing factors to the development of TMD [24]. Furthermore, TMD has been associated with migraine headaches and inflammatory disorders such as rheumatoid arthritis, juvenile idiopathic arthritis, and osteoarthritis [25].

Various methods have been used in orthodontic research to visualize changes in the treatment of temporomandibular joint resulting from functional treatment, such as cephalograms [26, 27], panoramic radiographs [28, 29], computed tomography [30, 31], and magnetic resonance imaging [32, 33]. However, image acquisition of the TMJ using conventional techniques is associated with several limitations.

CBCT scans provide accurate and precise quantitative data, allowing for comparisons of images without magnification and making them a valuable tool for analyzing treatment outcomes. These scans can also assist in volumetric measurements and can evaluate changes in the contours and forms of objects, which are often limited in 2D cephalometry. Moreover, CBCT scans provide more data than 2D images [34, 35]. In the presence of soft tissue, CBCT can reliably obtain volumetric and linear measurements of mandibular condyles [36]. However, only a few studies have investigated the TMJ’s positional and morphological characteristics and spaces in adults using 3D CBCT before and after treatments.

Based on the authors’ knowledge, this is the first study to evaluate the TMJ structure changes three-dimensionally following sequential molar distalization of the upper arch using clear aligners to correct class II malocclusion. Thus, this study aimed to three-dimensionally analyze the impact of sequential molar distalization using clear aligners on TMJ.

## Materials and methods

### Sample selection and procedure

**Fig. 1:**
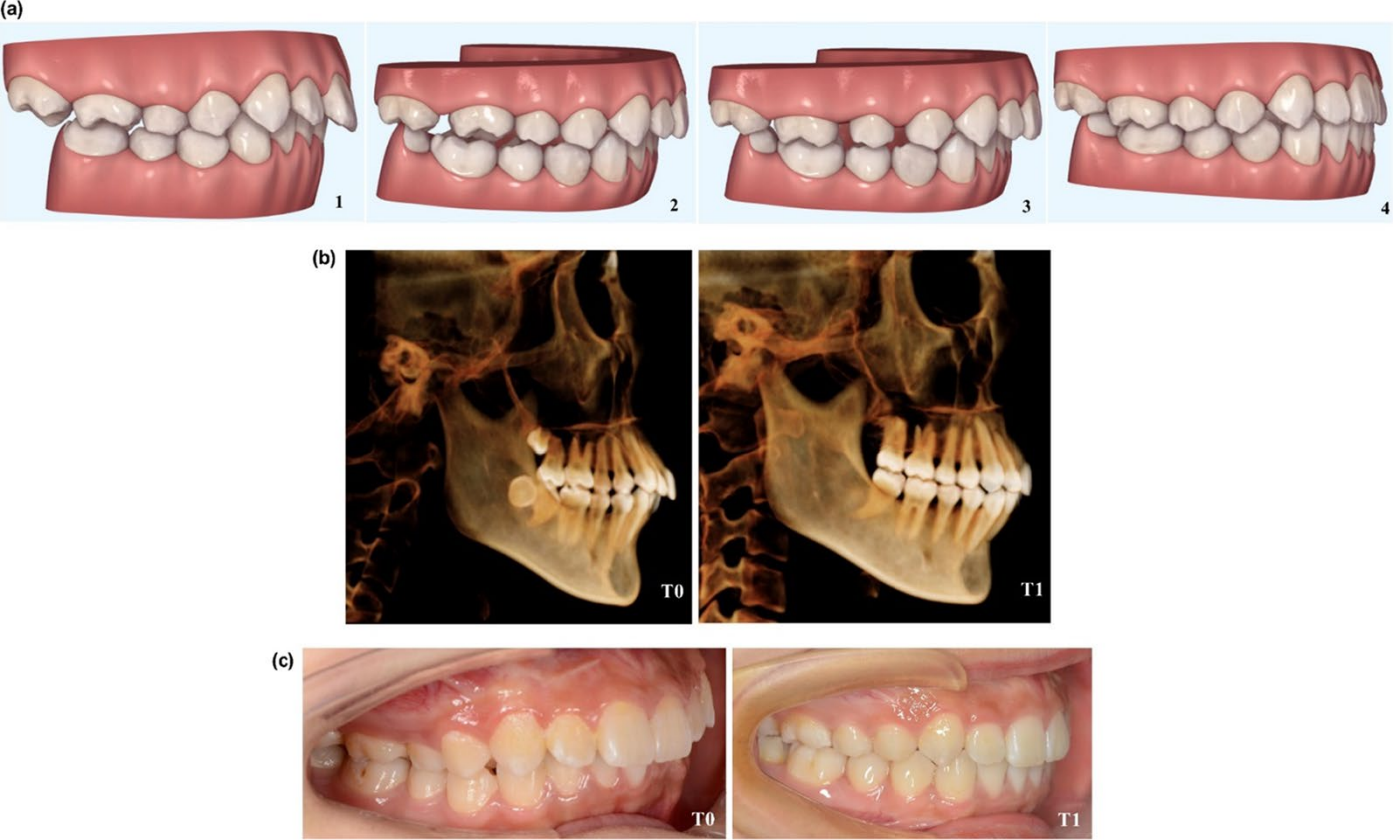
Illustrations for one of the treated patients. **a** Sequence of tooth movement with distalization of the upper molars, from (1 to 4). Figures extracted from ClinCheck® (Align Technology, San Josè, California, USA) **b** Lateral images extracted from the patient CBCT scan, before the orthodontic treatment with sequential distalization T0 and after treatment T1 **c** lateral intra-oral view of a patient before the orthodontic treatment T0 and after treatment T1

This retrospective study analyzed CBCT images of a sample of 23 non-growing subjects (16 females and 7 males; mean age 29.8 ± 4.6 years) treated with sequential molar distalization using orthodontic aligners (Invisalign, Align Technology, San Josè, California, USA). All procedures were conducted according to the Helsinki Declaration, and written consent forms were signed by all patients. Ethical approval was granted by the ethical committee of Lanzhou University’s School of Stomatology, Lanzhou, Gansu Province, China (ethical approval No. LZUKQ-2020-039). The sequential upper molars distalization treatment. Figure 1 was carried out by the same certified expert as suggested by Align Technology. The mean treatment time was of 23.6 ± 7.2 months. The achieved amount of maxillary molars distal movement in this study was an average of 2.54 mm and 2.18 for the first and second molars, respectively.

Inclusion criteria for all subjects were as follows: (1) over the age of 18, (2) with skeletal class I or class II malocclusion and a bilateral molar class II relationship, (3) all permanent teeth, except the third molar, have erupted, (4) no history of TMD symptoms in accordance with TMD Diagnostic Criteria [37], (5) good compliance during the treatment, (6) no prosthodontic or orthognathic treatment history, (7) and good definition and quality of the CBCT scans.

The exclusion criteria were as follows: (1) under the age of 18, (2) imaging manifestations of condylar degenerative conditions (e.g., condylar hyperplasia, subchondral cyst, and erosion), (3) extraction treatment except for third molars, (4) functional mandibular deviations or facial asymmetry, (5) surgical history at craniofacial region or TMJ, (6) any systemic disease or chronic medication use, (7) and skeletal malformation in the craniofacial region. Gender differences were not examined since only non-growing patients were involved in this study.

The sample size of the present study was estimated based on the study of (Caruso, Nota et al. 2019) using the G*Power 3.0.10. software (v3.1.9.7; Heinrich-Heine Universität Düsseldorf, Düsseldorf, Germany) depending on the molar relation (the primary outcome of this study). The a priori sample size calculation was performed with a power level of 95% at a 5% significance level (*α* = 0.05) and effect size (d*z* = 0.8), where the mean values of (MR) were 3.1 ± 1.4 and 1.2 ± 0.6 for pre- and post-treatment, respectively. The analysis indicated that at least 19 subjects are required. The sample size included in our study was 23 subjects.

The treatment protocol included the use of attachments that were placed following the attachment protocol of Align Technology to achieve predictable tooth movement [38], and the use of intermaxillary class II elastics. In addition, no adjunctive skeletal anchorage was used. Elastics were used while retracting the premolars, canines, and incisors to prevent the uncontrolled proclination of the anterior teeth and reinforce the anchorage [39].

### Cone-beam computed tomography (CBCT)

The I-CAT Imaging System (Imaging Sciences International Inc. Hatfield, USA) was used to execute CBCT. All patients were scanned with standard protocol: field of view (FOV) was 16.0 × 13.0 cm, the setting of exposure parameter was 18.54 MAs; 8.9 s; 120 kV, and image acquisition at 0.4 mm voxel size. Moreover, with head position standardization, Frankfort horizontal plane (FHP) parallel to the floor, and maximum occlusal intercuspation. According to the imaging protocol, the patients were asked to prevent from swallowing or moving throughout the scanning process.

### Three-dimensional measurement methods and the measured items

Digital Imaging and Communications in Medicine (DICOM) files of the CBCT images were obtained and then introduced into version 6.0.3 of the InVivoDental, (Anatomage Inc.) software program for the linear and angular three-dimensional and volumetric analysis.

The applied standard and innovative 3D TMJ analysis method was adopted from Alhammadi et al. [40–42] to measure the TMJ morphology-related parameters before and after receiving orthodontic treatment.

**Fig. 2:**
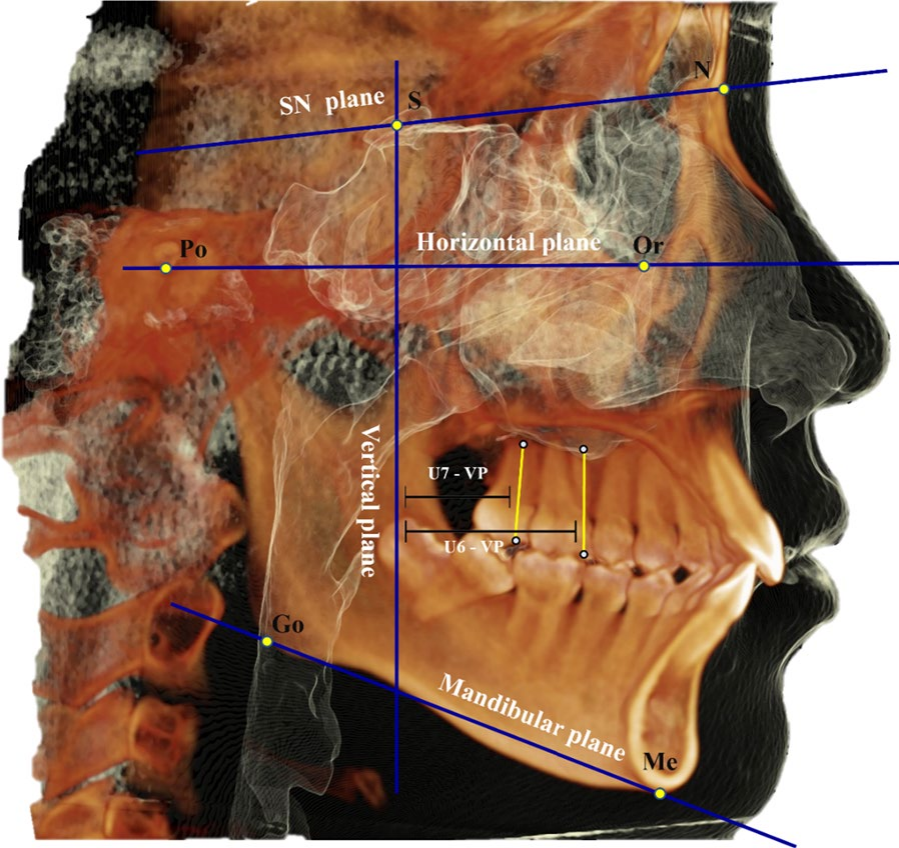
Craniofacial Landmarks and reference planes

**Fig. 3:**
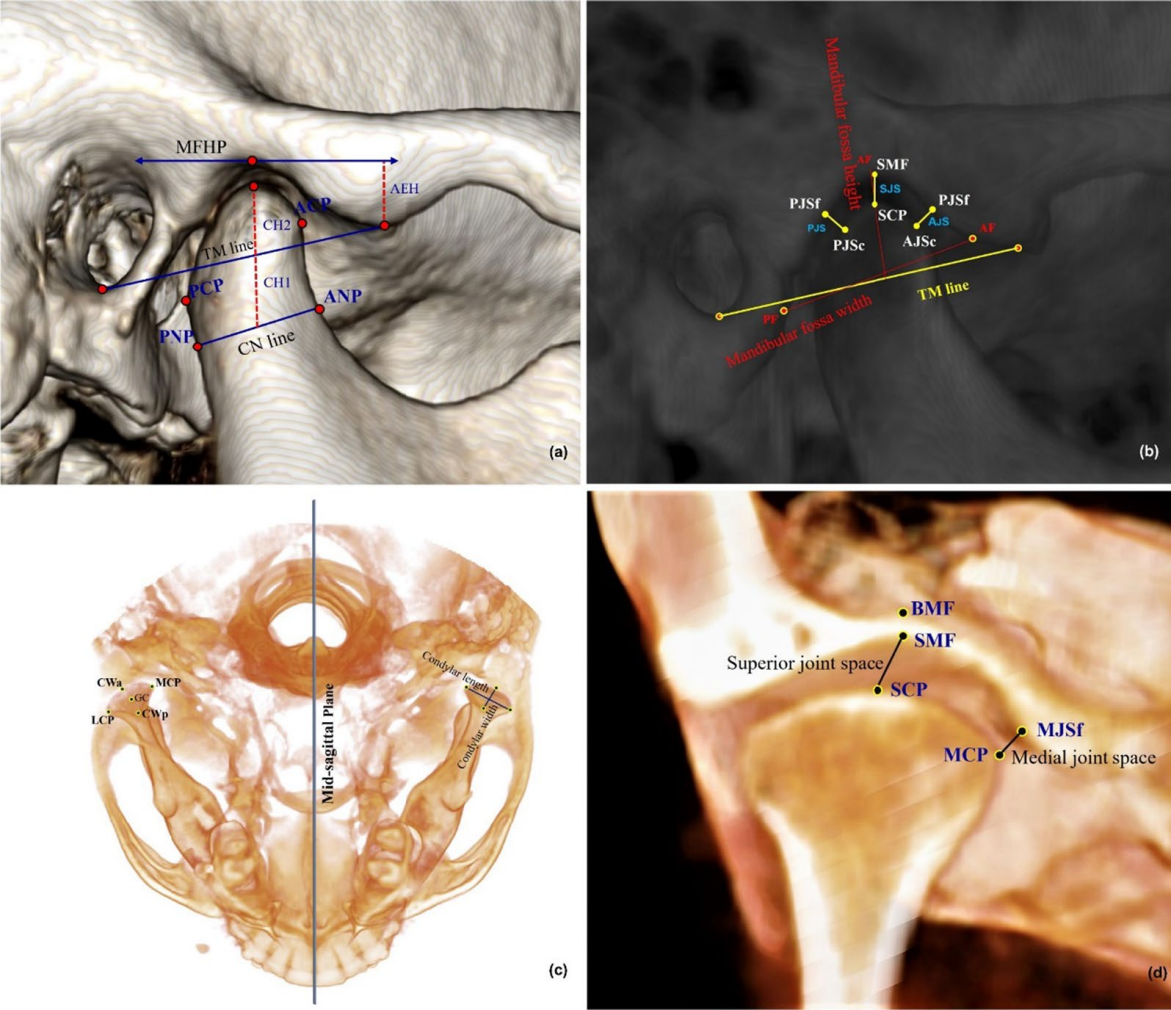
3D temporomandibular joint reference points and measurements: **a**, **b** sagittal views, **c** axial view, **d** coronal view

The 3D skeletal, dental, and TMJ landmarks are shown in Tables 1 and 2, respectively. The craniofacial reference planes, lines, and 3D measurements of TMJ are shown in Tables 3 and 4, respectively. Craniofacial reference planes are shown in Fig. 2, and the 3D TMJ reference points and measurements are shown in Fig. 3. On the basis of basal reference planes (MSP, HP, and VP), the condyle position was determined accurately and precisely in relation to the craniofacial structure.

**Table 1:** 3D skeletal and dental landmarks used in the study

Landmark	Abb	Definition
Nasion	N	The most anterior point of the frontonasal suture in the midsagittal plane
Sella	S	Midpoint of sella (the center of Sella turcica)
Basion	Ba	The lowest point on the anterior rim of the foramen magnum
Incisive Foramen	IF	The center of incisive foramen centered mediolateral, exists posterior to the central incisors at maxillary mid palatine
Orbital	Or	Lowest point on the inferior border of the orbit
Porion	Po	The most outer and superior bony point of the external auditory meatus
Gonion	Go	The point of bisecting angle connecting the ramus line and body of the mandible line
Menton	Me	The most inferior midpoint of the chin on the mandibular symphysis outline
Pogonion	Pog	The most anterior point on the mandibular symphysis
Subspinale	A	The most posterior concave point at the middle of the frontal maxilla
Supramental	B	The most posterior concave point at the middle mandibular symphysis process
U6_Cusp	U6C	The mesiobuccal cusp tip of upper first molar
U6_Apex	U6A	The mesial root apex of upper first molar
U7_Cusp	U7C	The mesiobuccal cusp tip of upper second molar
U7_Apex	U7A	The mesiobuccal root apex of upper second molar
L6_Cusp	L6C	The mesiobuccal cusp tip of lower first molar
L6_Apex	L6A	The mesial root apex of lower first molar
L7_Cusp	L7C	The mesiobuccal cusp tip of lower second molar
L7_Apex	L7A	The mesiobuccal root apex of lower second molar

**Table 2:** 3D TMJ landmarks used in the study

	Landmark	Abb	Definition
Coronal view	Soft tissue mandibular fossa	SMF	The middlemost and highest point of the soft tissue mandibular fossa
	Bony mandibular fossa	BMF	The middlemost and highest point of the bony mandibular fossa
	Medial joint space “fossa point”	MJSf	The most lateral point of the mandibular fossa medial wall
	Superior condylar point	SCP	The most top point of the condylar head
	Medial condylar point	MCP	The most medial point of the condylar head
Axial view	Lateral condylar point	LCP	The most lateral point of the condylar head
	Condyle Geometric center	GC	Approximately centered mediolaterally and anteroposterior and respectively from all views
	Condyle width “anterior point”	CWa	Axially, most anterior prominent point of condyle head at the region with the greatest width
	Condyle width “posterior point”	CWp	Axially, most posterior prominent point of condyle head at the region with the greatest width
Sagittal view	Anterior condylar point	ACP	The sagittal most prominent point anteriorly of the condylar head
	Posterior condylar point	PCP	The sagittal most prominent point posteriorly of the condylar head
	Articular tubercle	AT	The most inferior point of the anterior tubercle
	Inferior meatus	IM	The most inferior point of the external auditory meatus
	Anterior fossa	AF	The most anterior and inferior point in the inner anterior wall of the glenoid fossa
	Posterior fossa	PF	The most posterior and inferior point in the inner posterior wall of the glenoid fossa, which in parallel line with IM
	Anterior condyle neck point	ANP	The deepest point at the anterior concave wall of condylar neck
	Posterior condyle neck point	PNP	The deepest point at the posterior concave wall of condylar neck approximately at the parallel line with ANP
	Anterior joint space “mandibular fossa point”	AJSf	The most prominent posterior point of the anterior inner wall of glenoid fossa opposed to the closest anterior condyle-fossa distance
	Anterior joint space “condylar point”	AJSc	The most prominent anterior point of posterior inner wall of glenoid fossa opposed to the closest anterior condyle-fossa distance
	Posterior joint space “mandibular fossa point”	PJSf	The most prominent anterior point of the posterior inner wall of glenoid fossa opposed to the closest posterior condyle-fossa distance
	Posterior joint space “condylar point”	PJSc	The most prominent posterior condyle head point opposed to the closest posterior condyle-fossa distance

**Table 3:** The reference planes and lines used in the study

Reference plane/line	Abb	Definition
Horizontal plane	HP	Constructed by three points right orbital with two side porion
Midsagittal plane	MSP	Constructed by three points N, BA, and IF
Vertical plane	VP	Constructed of sella point and perpendicular to the midsagittal and horizontal plane
Mandibular plane	MP	Constructed by three points; right, left gonion and menton
Mandibular fossa horizontal plane	MFHP	Tangent to the right and left BMF separately and parallel to the horizontal plane
TM line	TML	Determined through auditory meatus and anterior tubercle
Mandibular fossa line	MFL	Determined through the two bony mandibular fossae points BMF
Anteroposterior condylar line	ACP–PCP	A line extended from ACP to PCP
Mediolateral condylar line	MCP–LCP	A line extended from MCP to LCD
Sagittal condylar neckline	CNL	A line extended from ANP–PNP
Upper first molar long axis	U6	The long axis of upper first right or left molar extending from U6_cusp to U6_apex
Upper second molar long axis	U7	The long axis of upper second right or left molar extending from U7_cusp to U7_apex
Lower first molar long axis	L6	The long axis of lower first right or left molar extending from L6_cusp to L6_apex
Lower second molar long axis	L7	The long axis of lower second right or left molar extending from L7_cusp to L7_apex

**Table 4:** 3D measurements used in the study

Measurement	Abb	Definition
*Skeletal measurements*		
Maxillary anteroposterior position	SNA	The angle formed between 3-point landmarks; sella, nasion, and A points
Mandibular anteroposterior position	SNB	The angle formed between 3-point landmarks; sella, nasion, and B points
Skeletal anteroposterior jaw relation	ANB	The angle formed between 3-point landmarks; A point, N point, and B point
Skeletal vertical jaw relation	MP^SN	The angle between sella-nasion (SN) and Go-Me (MP)
*Dental measurements*		
U6—VP	The distance from upper first molar cusp tip to VP, measuring the amount of first molars distalization	
U7—VP	The distance from upper first molar cusp tip to VP, measuring the amount of second molars distalization	
L6—VP	The distance from lower first molar cusp tip to VP, measuring the amount of first molars mesialization	
L7—VP	The distance from lower first molar cusp tip to VP, measuring the amount of second molars mesialization	
*Mandibular fossa dimension*		
Mandibular fossa height	MFH	Distance extends perpendicularly between BMF and TM line
Mandibular fossa width	MFW	Distance extends horizontally between AF and PF
Articular eminence height	AEH	The perpendicular distance between AT and MFHP
*Condylar inclination*		
Mediolateral condylar inclination	MCI	Angle between MCP-LCP line and HP
Vertical condylar inclination	VCI	Angle between ACP-PCP line and VP plane
Anteroposterior condylar inclination	APCI	Angle between MCP-LCP line and MSP
*Condylar position*		
Vertical condylar position	VCP	Distance extends vertically from SCP to HP
Anteroposterior condylar position	APCP	Distance extends anteroposterior from ACP to VP
Mediolateral condylar position	MLCP	Distance extends mediolaterally from MCP to MSP
Vertical condylar joint position	VCJP	Linear difference between condyle height to TM line and condyle height to the condyle neckline
*Condylar dimension*		
Condylar length	CL	The mediolateral distance from MCP to LCP
Condylar width	CW	The anteroposterior condylar width CWa to CWp
Condylar height	CH1	The perpendicular distance from SCP to CN line (ANP-PNP)
Condylar height to TM line	CH2	The perpendicular distance from SCP to TM line
*TMJ spaces*		
Anterior joint space	AJS	Closest distance between AJSc and AJSf
Posterior joint space	PJS	Closest distance between PJSc and PJSf
Superior joint space	SJS	Closest distance between SCP and SMF
Medial joint space	MJS	Closest distance between MCP and MJSf
Volumetric total joint space (mm^3^)	VTJS	Total volumetric mandibular joint spaces (superior, anterior, and posterior) which enclosed by TM line

**Fig. 4:**
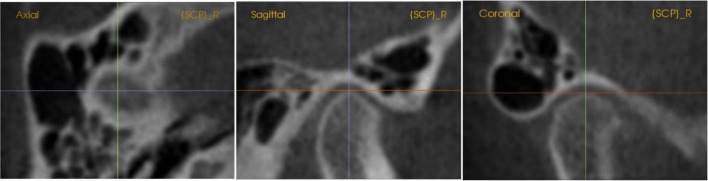
Slice locator of 3D landmarks determination

The 3D analysis was designed based on the determination points in this sequence. First, the coordinate system’s orientation is set according to facial skeletal points of midline: nasion, basion, and incisive foramen, which were proved as valid points by Green et al. [43], and the lateral landmarks determined by orbital and porion points. Secondly, the landmarks were digitized based on which were the most identified and obvious in the 3D image. Then, the position of each traced point was adjusted by the slice locator on each of the three planes individually, as shown in Fig. 4.

**Fig. 5:**
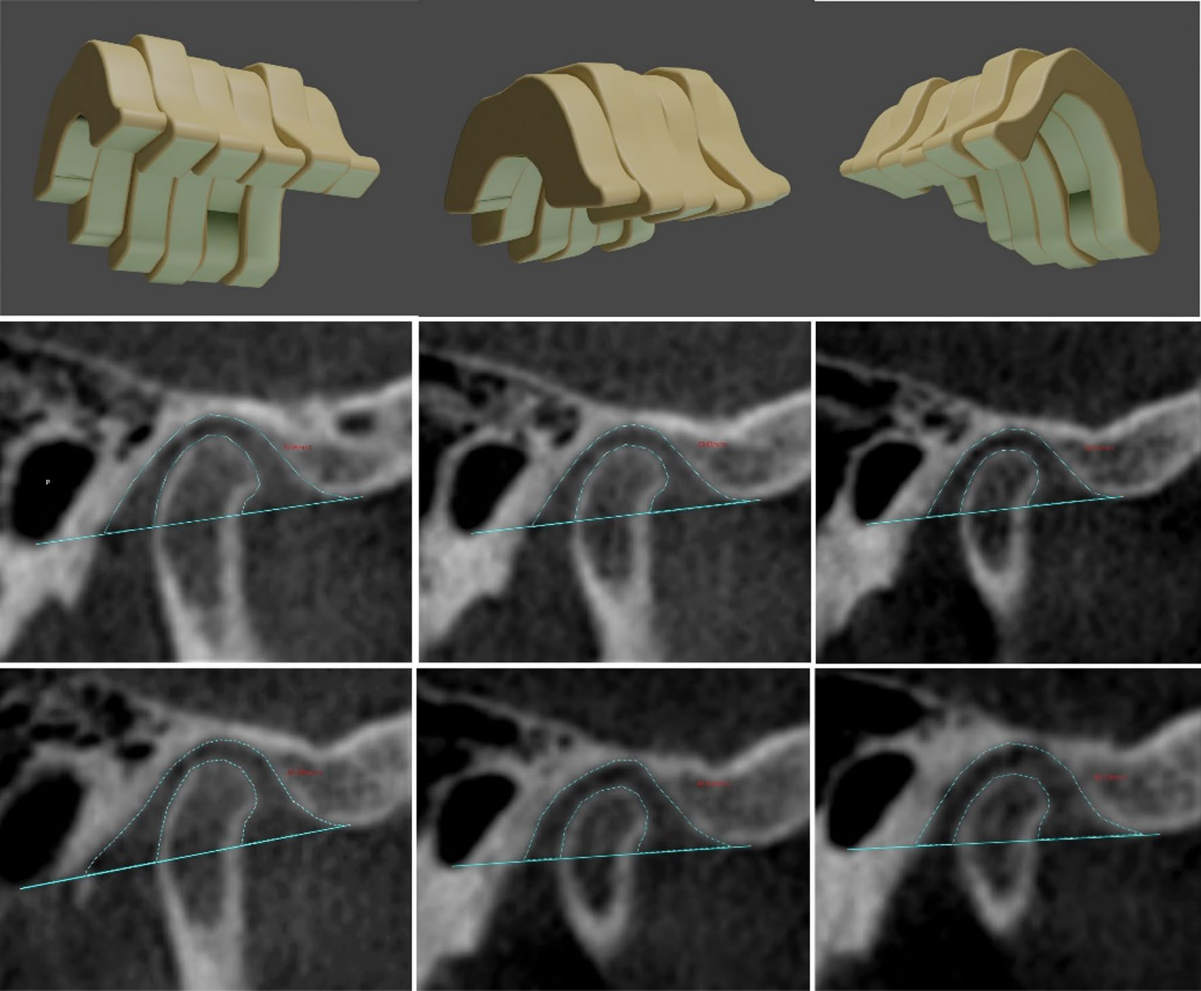
3D volumetric total joint space with 2D identification points

A prior study by Abdulqader et al. [41] was published regarding the volumetric joint space analysis. We followed their approach of measuring the volumetric TMJ space by cubic 3D analysis of the whole joint space by sectioning the total joint space into six sections for each side, as in Fig. 5, and each section had a 1.5 mm width with the entire surface area. Then, spaces were then calculated using the sigma volume formula ${\varvec{v}} \cong \sum\nolimits_{{{\varvec{k}} = 1}} {{\varvec{A}}\left( {{\varvec{x}}_{{\dot{\user2{I}}}} } \right)\Delta {\varvec{x}}}$. All variables on both sides were measured to eliminate any probable improperness of the left and right side differences.

### Intra- and inter-observer method error

For method error verification, the intra- and inter-examiner reliability of the measurements was analyzed by retracing 10 cases by two different observers within 2 weeks. Intra-class correlation coefficients (ICCs) and the absolute and relative technical error of measurement (TEM and rTEM) were calculated to determine the reliability and reproducibility of the measurements. Bland–Altman plot was also used to assess the reproducibility and reliability of TMJ landmarks (see additional file 1).

### Statistical analysis

Version 27 of the SPSS Statistics software (IBM Corp., Armonk, NY, USA) was used for statistical analysis. GraphPad Prism 8 was used to plot the graphs. Data were checked for normal distribution using the Shapiro–Wilk test. For each variable of 46 joints, descriptive statistics of the mean and standard deviation (SD) were calculated. The paired T test was employed to examine the differences between the two sides’ TMJ parameters before (T0) and after (T1) molar distalization periods. The significance level was chosen at *P* < 0.05.

## Results

Descriptive data among the 23 adult patients with class II malocclusions fulfilled the inclusion and exclusion criteria. The frequency of molar relationship Class II regarding its severity was as follows: six subjects 1/4 cusp, nine subjects 1/2 cusp (end-to-end), and eight subjects full cusp Class II relationship. In addition, 7 and 16 subjects were males and females, respectively, aged 22–47 years, with an average age of 29.8 ± 4.6 years. The means and standard deviation for each variable of recorded data are presented in Table 5. Figure 1 shows the sequence of upper molars distalization movement extracted from ClinCheck® and the lateral radiological and clinical intra-oral views before and after treatment for one of the treated patients.

**Table 5:** Descriptive data and statistical analysis of the dental, skeletal, and TMJ parameters between T0 and T1

Measurements	T0		T1		T-test
	Mean	SD	Mean	SD	
*Skeletal measurements*					
SNA	82.32	0.11	81.99	0.19	0.261
SNB	80.12	0.59	79.74	0.61	0.696
ANB	2.20	0.48	2.25	0.43	0.940
MP^SN	36.39	1.46	36.70	2.08	0.913
*Dental measurements*					
U6—VP	18.85	1.16	16.31	1.28	0.032*
U7—VP	9.90	1.21	7.72	0.98	0.003**
MR	3.29	0.32	1.10	0.38	0.000***
L6—VP	17.40	0.48	17.65	0.32	0.71
L7—VP	8.88	0.13	9.09	0.14	0.39
*Mandibular fossa dimension*					
MFH	8.19	1.07	7.75	1.08	0.413
MFW	16.17	1.42	18.78	1.93	0.375
AEH	7.87	1.18	8.05	1.20	0.889
*Condylar inclination*					
MCI	26.38	7.61	24.34	9.57	0.890
VCI	7.04	1.60	7.94	1.98	0.765
APCI	76.86	4.97	76.88	3.99	0.815
*Condylar position*					
VCP	1.71	0.77	1.55	0.53	0.851
APCP	7.34	2.15	7.15	1.55	0.613
MLCP	53.24	2.51	52.66	2.39	0.553
VCJP	5.64	0.45	5.91	0.33	0.425
*Condylar dimension*					
CL	18.94	1.16	19.06	1.06	0.946
CW	10.01	0.79	9.27	0.18	0.388
CH1	11.75	0.74	12.03	1.12	0.666
CH2	5.82	1.12	6.21	0.66	0.642
*TMJ spaces*					
AJS	2.68	1.16	2.78	1.06	0.918
PJS	2.73	1.31	1.79	0.19	0.267
SJS	2.63	0.22	3.19	0.02	0.252
MJS	4.67	0.92	4.36	0.66	0.650
VTJS (mm^3^)	375.06	17.15	358.70	18.98	0.310

^*^P < 0.05; **P < 0.01; ***P < 0.0001

**Fig. 6:**
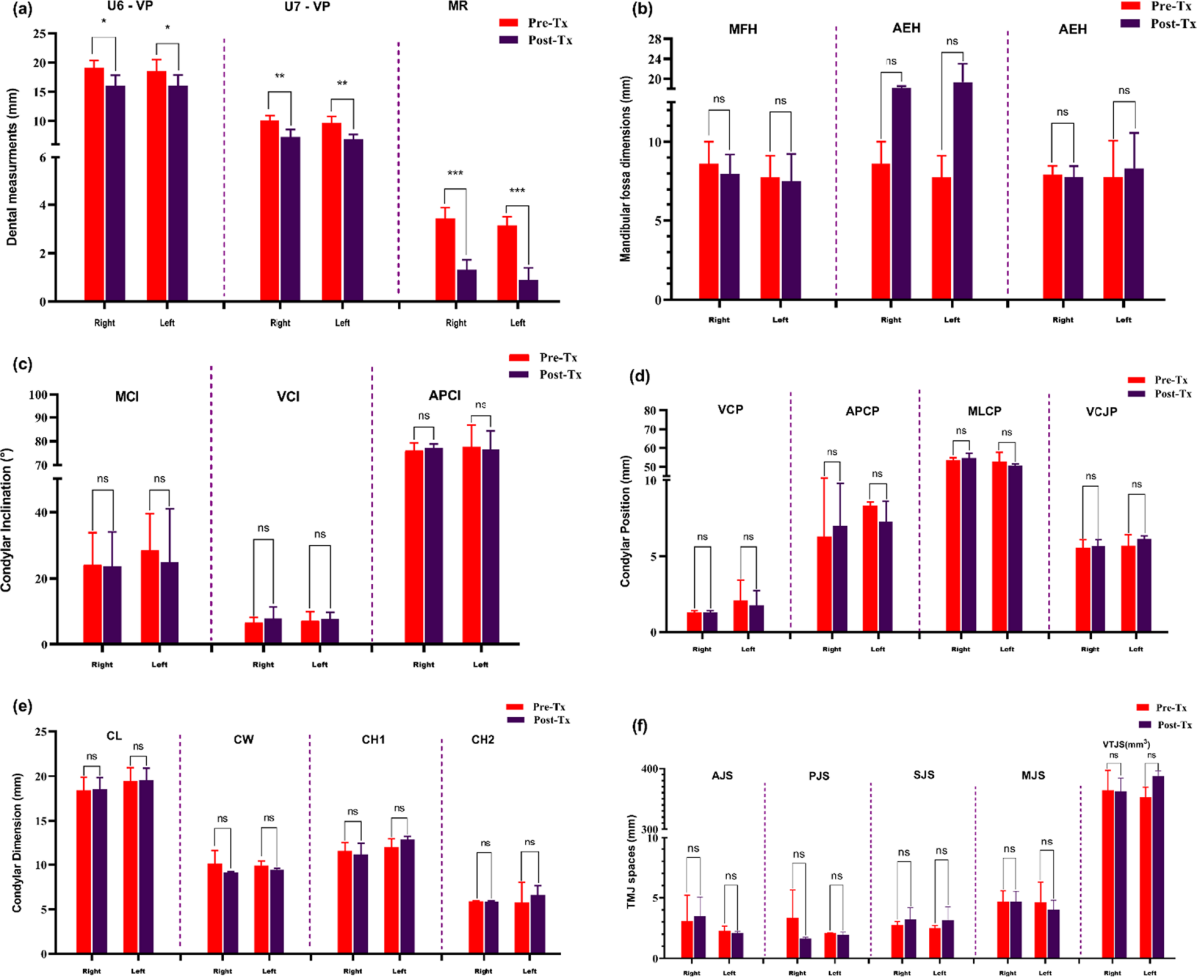
**a**–**f** Statistical graphs of the differences between the right and left TMJ measurements between T0 and T1. *P < 0.05; **P < 0.01; ***P < 0.0001; *ns* non-significant

No statistically significant differences were found in the net impacts resulting from maxillary molars distalization by clear aligners on the osseous mandibular joint’s components and joint spaces on both left and right sides of patients before and after treatment (all *P* > 0.05), as shown in Fig. 6. Meanwhile, a significant clinical improvement was observed in the molars relation (MR). Regarding the dentoalveolar measurements, the first and second maxillary molars positions were significantly reduced (*P* ≤ 0.03) by 2.54 and 2.18 mm, respectively, as shown in Fig. 6a.

For the mandibular fossa (MF) dimensions, no statistically significant differences were found between T0 and T1. Moreover, no statistically significant changes were found for the mandibular condyle inclination, position, and dimension between before and after maxillary molar distalization of the treated sample. Similarly, no statistically significant variations were observed for the analyzed volumetric total joint space and other TMJ space measures.

Intra- and inter-observer reliabilities analysis data of all the TMJ measurements are presented in Table 6, showing an excellent correlation. Bland–Altman analysis demonstrated very good intra- and inter-observer agreement between X, Y, and Z coordinates for all TMJ landmarks (Additional file 1: Appendix A).

**Table 6:** Results of intra-class correlation coefficient (ICC) reliability analysis of the 3D measurements used in the study

Measurements	Intra-observer				Inter-observer			
	ICC	TEM	rTEM	R	ICC	TEM	rTEM	R
MFH	0.982	0.31	2.48	0.982	0.962	0.24	1.98	0.962
MFW	0.977	0.41	2.21	0.977	0.964	0.38	2.16	0.966
AEH	0.971	0.34	2.46	0.981	0.982	0.34	2.41	0.978
MCI	0.971	0.49	5.28	0.971	0.947	0.48	4.92	0.947
VCI	0.976	1.02	1.67	0.976	0.949	1.04	1.63	0.946
APCI	0.963	1.04	1.89	0.963	0.989	1.16	2.12	0.989
VCP	0.970	0.04	16.27	0.968	0.977	0.20	15.75	0.977
APCP	0.965	0.31	4.27	0.965	0.966	0.31	4.30	0.966
MLCP	0.960	0.48	0.89	0.960	0.979	0.48	0.92	0.979
VCJP	0.975	0.25	4.04	0.975	0.969	0.23	4.11	0.969
CL	0.971	0.42	2.48	0.971	0.957	0.45	2.10	0.957
CW	0.979	0.26	3.71	0.979	0.958	0.25	3.21	0.958
CH	0.978	0.35	3.14	0.978	0.962	0.35	3.32	0.962
CH2	0.969	0.32	3.26	0.965	0.964	0.34	3.31	0.962
AJS	0.980	0.05	2.91	0.978	0.986	0.06	2.91	0.986
PJS	0.980	0.09	2.57	0.969	0.967	0.07	2.65	0.963
SJS	0.971	0.19	6.16	0.971	0.965	0.19	6.17	0.965
MJS	0.968	0.17	5.19	0.968	0.968	0.16	5.77	0.965
VJS	0.968	6.98	2.52	0.968	0.970	6.92	2.43	0.970

## Discussion

The TMJ pretreatment values could be used to assess TMJ changes and evaluate treatment outcomes after orthodontic or orthognathic treatment in young adults. The detailed measurements of the TMJ’s anatomical structures in three-dimensional planes of their interpretations will help understand TMJ’s pathological alterations [44].

The excellent correlation coefficient between intra- and inter-observer reliability measurements indicated high and precise landmark identification with CBCT, which is regarded as an ideal tool for osseous assessment of the anatomic structures and cannot be obtained with any other conventional modality used to evaluate the complex temporomandibular region [45, 46].

As the first study that three-dimensionally evaluated the TMJ structure changes using CBCT before and after sequential molar distalization of the upper arch by clear aligners for correcting class II malocclusion, the result of this study will be helpful in clinical treatment planning for asymptomatic patients or patients with subjective TMD symptoms who proposed to undergo orthodontic treatment.

In an adult, class II correction mainly results from tooth movement without the effects of growth, and molar distalization is often undertaken to gain 2 to 3 mm of space in the dental arch in order to obtain a class I relationship [47]. In class II malocclusions, upper third molars, if present, should be removed to provide sufficient space for the movement of the first and second molars [48].

Results indicated the potential of maxillary molar bodily movement, at least when a minimal sagittal plane correction is needed, whereas our sample included subjects with multiple complexities of Class II molar relations varied from 1/4 cusp to full cusp of Class II molar relationship. A significant distal movement was observed of the upper molars and the related correction in the molar relationship (MR) with the absence of changes in mandibular fossa dimension and condylar inclination, position, and dimension outcomes for pre- and post-molar distal movement, thereby confirming the capability of performing a distal body movement of the upper molars by clear aligners with complete control of the TMJ measures the opposite of what has been reported with other orthodontic appliances [49]. The position and movement of lower molars from T0 to T1 were also evaluated. The results indicated no significant changes in the mandibular molars' position during Class II correction. This confirms that lower molars were not involved in mesialization during the treatment.

Furthermore, no significant change has been demonstrated regarding the mandibular fossa dimension. Similarly, no significant differences were observed between the pre-and post-treatment groups in condylar inclination and condylar dimension for both sides after correcting class II malocclusion by Invisalign aligners.

Anterior or posterior condyle position may directly affect facial morphology [50, 51]. In our study, the condylar position was examined using two distinct approaches Fig. 6d. The first approach relied on dependent planes (MSP, HP, and VP). Regarding the anteroposterior condylar position relative to the vertical plane, this study showed no clinically important differences between T0 and T1. In addition, regarding the vertical condylar position relative to the horizontal plane, the distalization movement with aligners was not associated with a significant superior condylar position after treatment. The second approach relies on establishing the concentric position of the mandibular condyle in the glenoid fossa using the Pullinger and Hollender formula [52] to obtain the ratio between the anterior and posterior joint spaces. The current study showed a statistically non-significant ratio of condylar joint position between T0 and T1. This indicates that the condyles are in the same position after treatment. Thus, the mandibular condyles seem to be concentric to their articular fossae.

Lione et al. reported that clear aligners provide better vertical dimension control during distal teeth movement. The thickness of aligners and the impact of the biting block of aligner material may explain the nonexistence of a significant vertical dimension increase [53]. The insignificance of our finding could be interpreted as molar distalization by clear aligners associated with the absence of molar extrusion and clockwise rotation of the occlusal plane in contrast with conventional appliances [11]. This would lead to premature contact and sudden alteration of temporomandibular components’ relation.

As for TMJ spaces, no statistically significant differences were observed in both sides of anterior, posterior, superior, and medial joint spaces before and after the treatment by distalization of upper molar teeth with clear aligners. However, volumetric joint space effects of molar class II correction with aligners have never been described [49]. The present study showed non-significant variations in TMJ spaces, and a slight increase of VTJS mean in T1 as compared with that in T0 Fig. 6f, thereby indicating no reduction in condylar dimensions. Most common changes in the morphology of the mandibular condyle, such as decreased volume, are indicative of TMD [54]. Clinically, this assessment can be used to diagnose TMJ in patients suffering from malocclusion with no symptoms of pain or TMJ dysfunction [55].

Looking at the results of this study, the upper molars distalization technique performed with clear aligners seems to overcome various side effects related to this orthodontic procedure typically observed with other appliances [11, 49] and seems to allow a predictable distal body movement of upper molars with control of the TMJ parameters. This could be related to the aligner design, which enables the control of 3D movements by holding teeth on all the surfaces (occlusal, vestibular, and palatal/lingual) and applying proper forces thanks to properly digitally planned attachments.

Accordingly, orthodontic aligners could represent an effective option for molar distalization approach, especially for TMJ pathologies subjects, at least for molar distal movements up to 2–3 mm.

This work is limited by its low sample size. In future studies, the mean amount of distal movement should be increased with various groups of malocclusion comparison to validate the control of TMJ structures after molar distalization using clear aligners.

## Conclusion

The study revealed insignificant changes in condyle-fossa spaces, dimension, and position in patients treated for class II malocclusion with sequential molar distalization using clear aligners, indicating that clear aligners do not significantly impact TMJ parameters during or after sequential molar distalization. Accordingly, sequential molar distalization using clear aligners is a viable treatment option for class II malocclusion patients without adversely affecting TMJ parameters. However, orthodontists need to consider the effects of various orthodontics appliances on TMJ components when prescribing treatment for their patients.

## Supplementary Information

**Additional file 1. Appendix A:** Supplementary data.

## Data Availability

Not applicable.
