# Supplementary material for: Impact of molar teeth distalization by clear aligners on temporomandibular joint: a three-dimensional study
Source: Prog Orthod. 2023 Jul 17;24:25. doi: 10.1186/s40510-023-00474-3 (PMC10350446; doi:10.1186/s40510-023-00474-3)

Impact of molar teeth distalization by clear aligners on temporomandibular joint: A three-dimensional study

Appendix A Supplementary data:

Bland-Altman plots in Intra and Inter-observer reliability for the TMJ landmarks

Bland Altman Plot for "SMF Point"

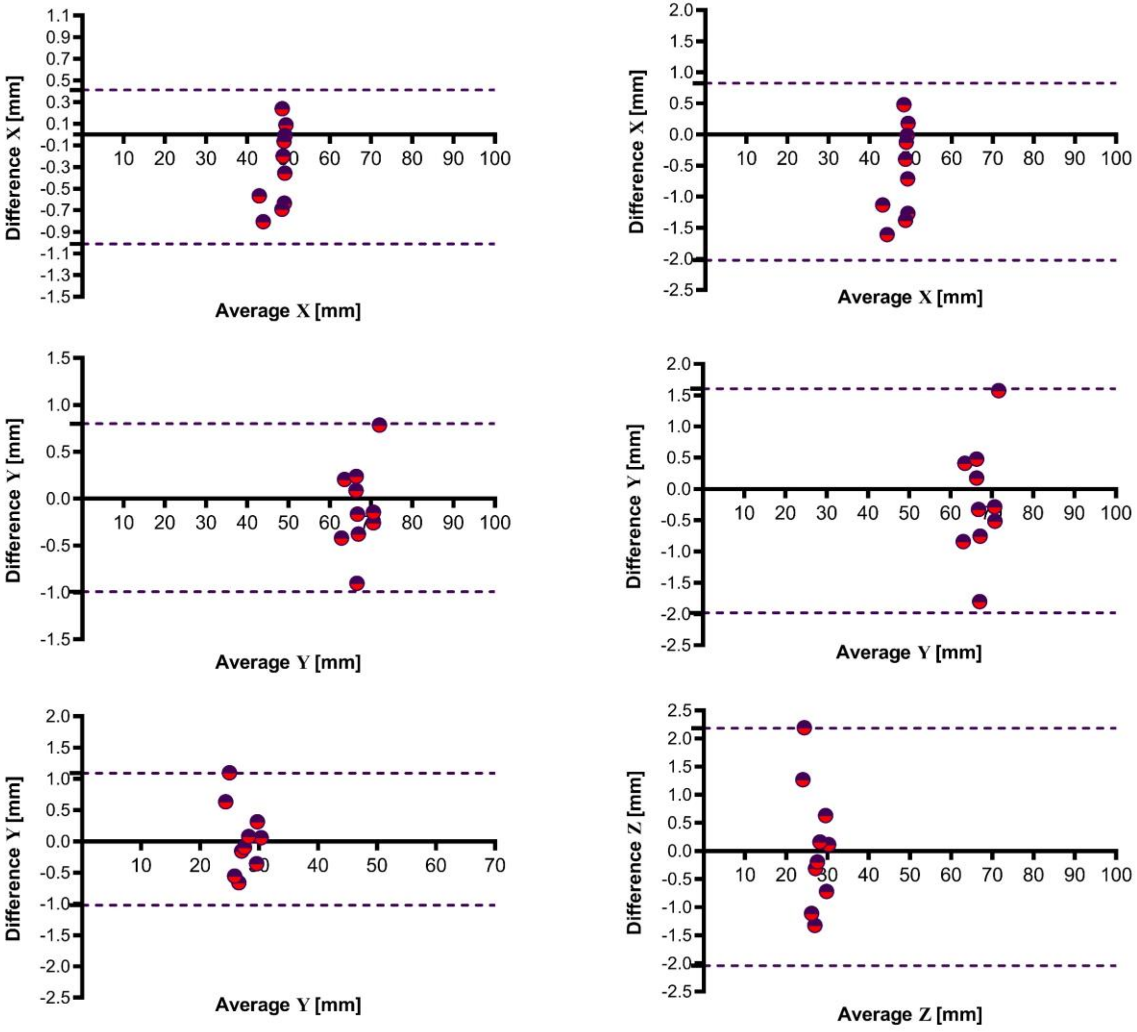

Bland Altman Plot for "BMF Point"

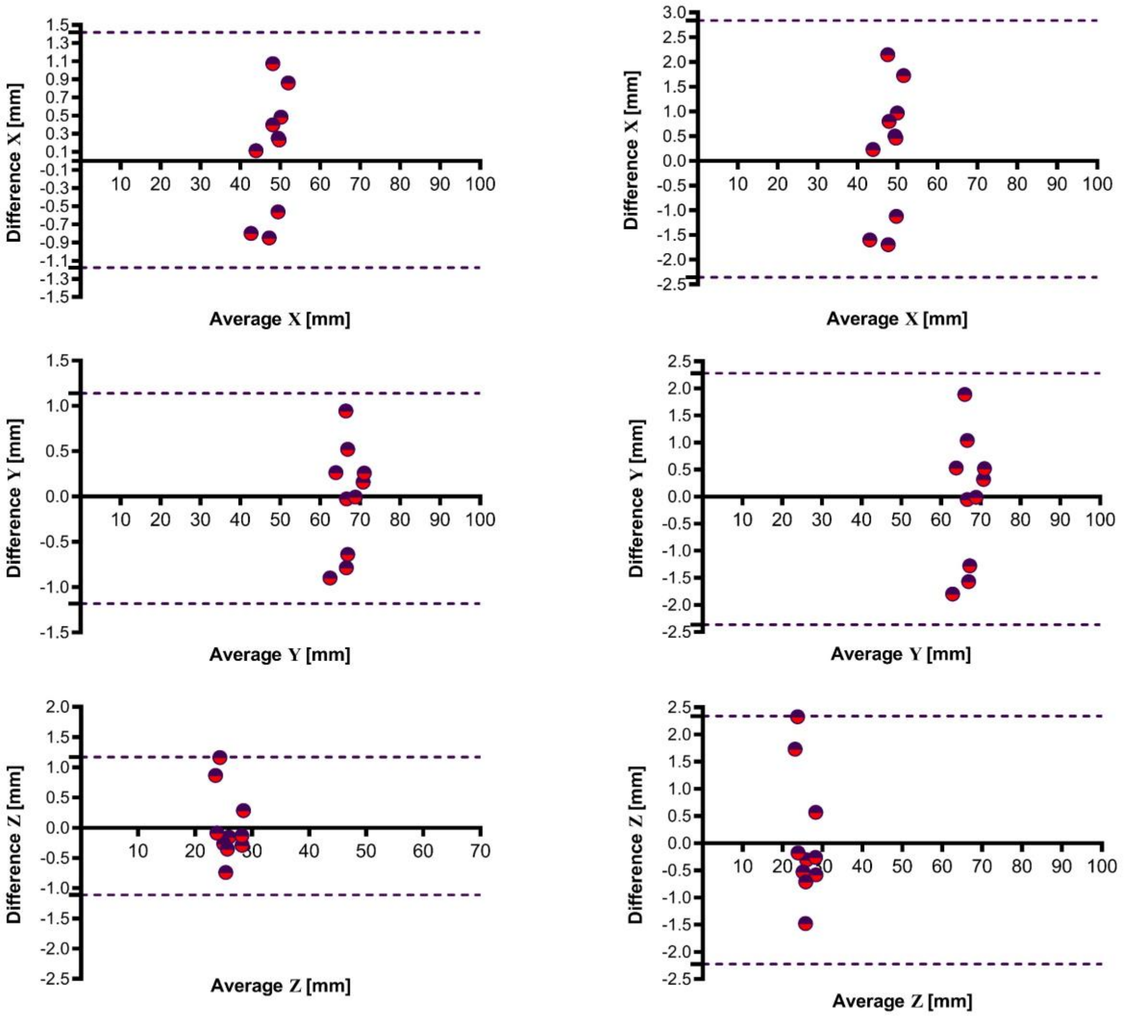

Bland Altman Plot for "MJSf Point"

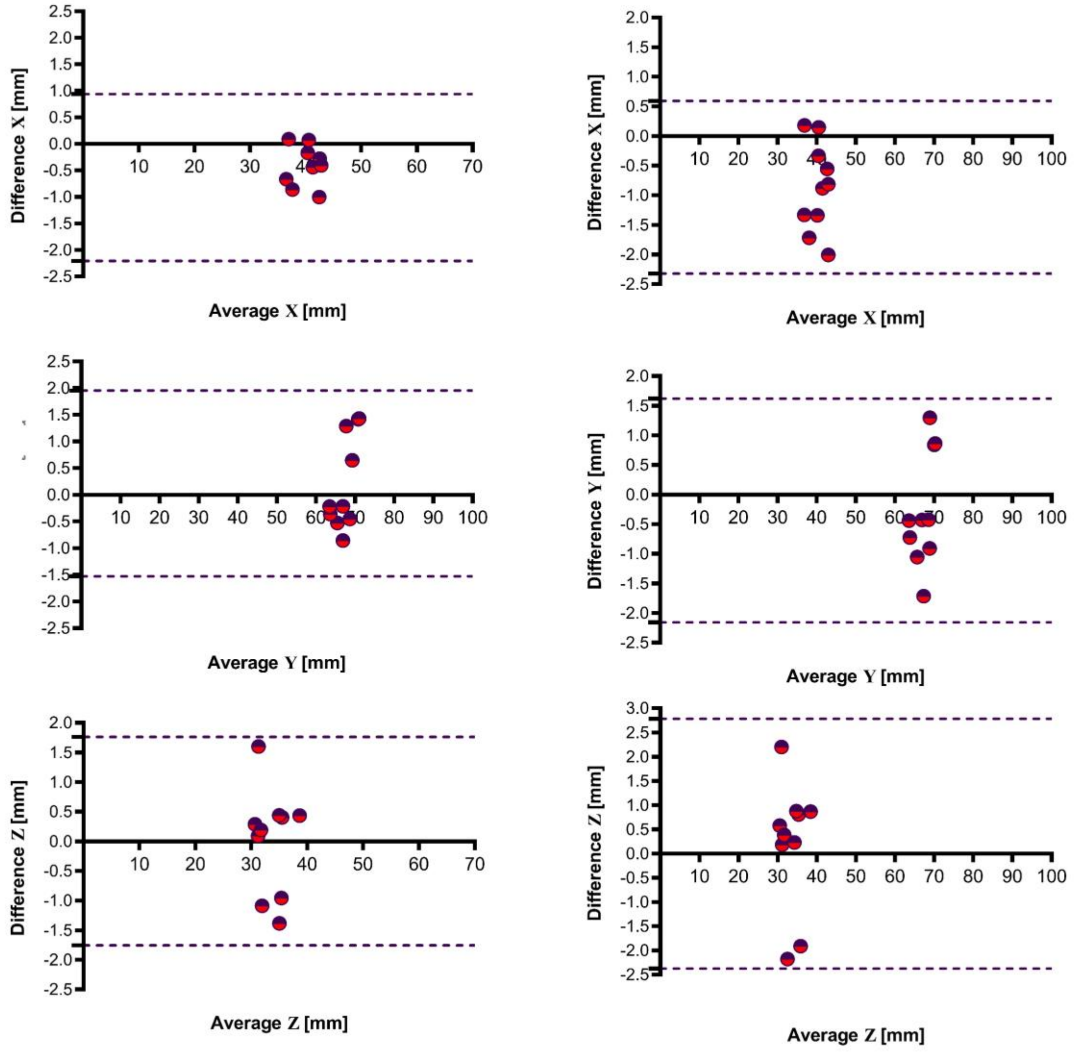

Bland Altman Plot for "SCP"

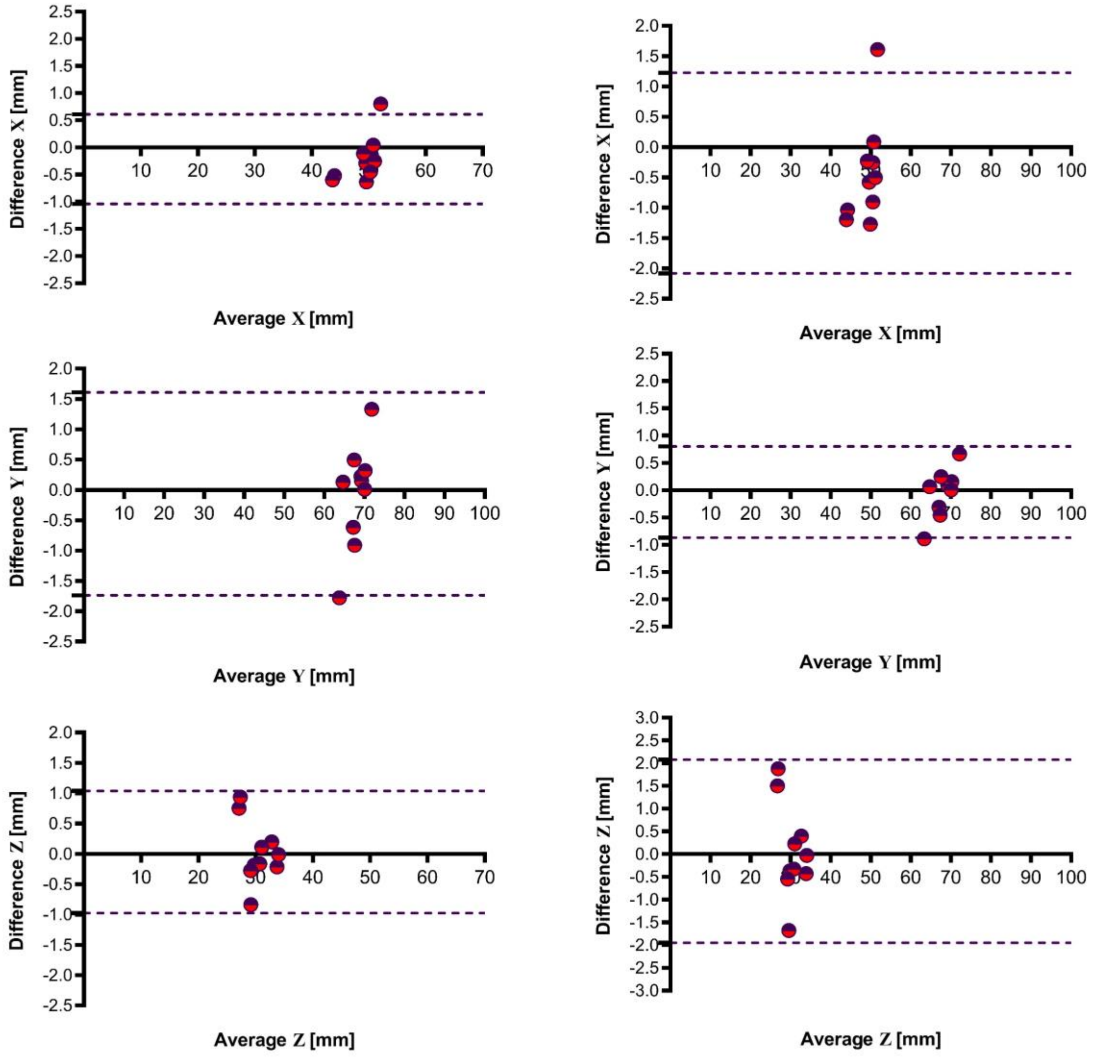

Bland Altman Plot for "MCP"

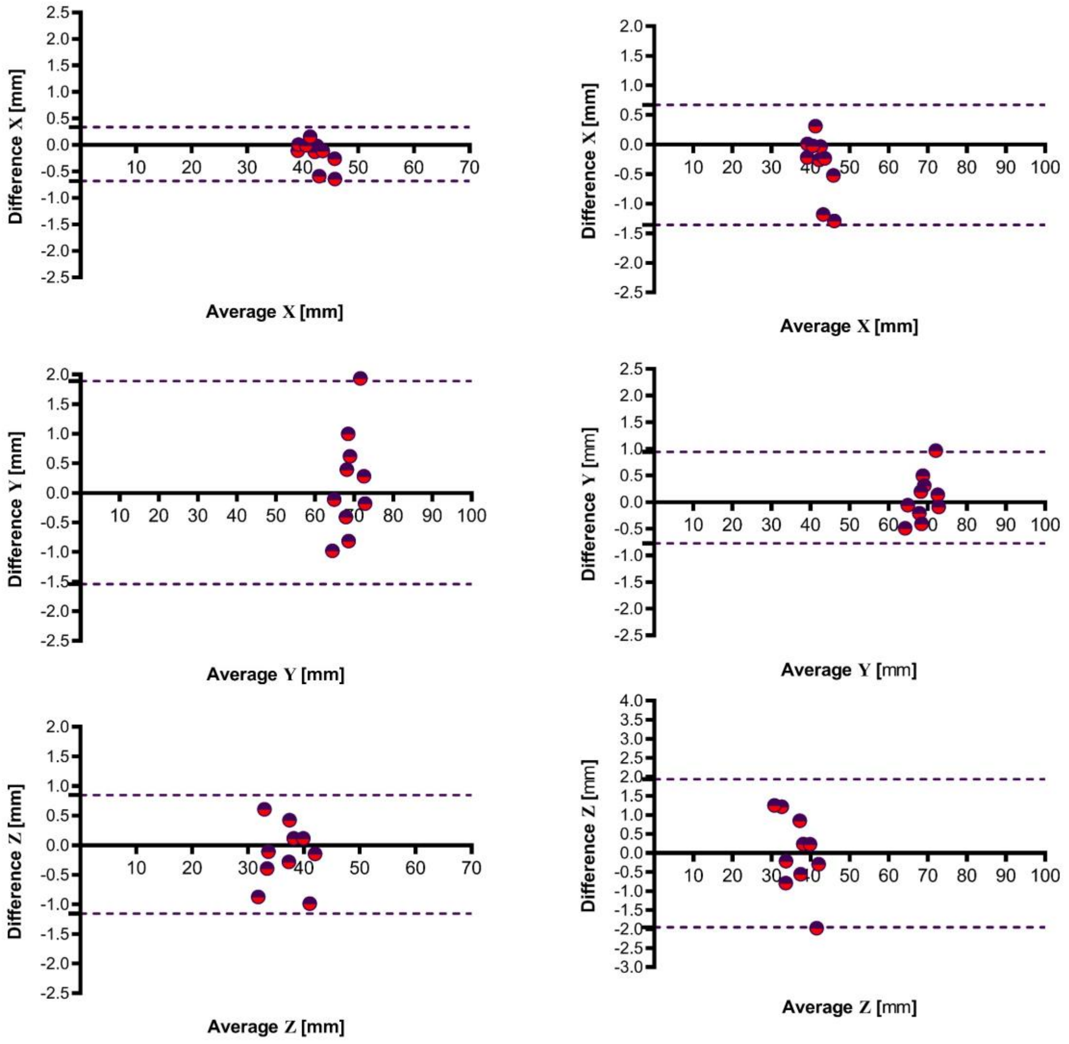

Bland Altman Plot for "LCP"

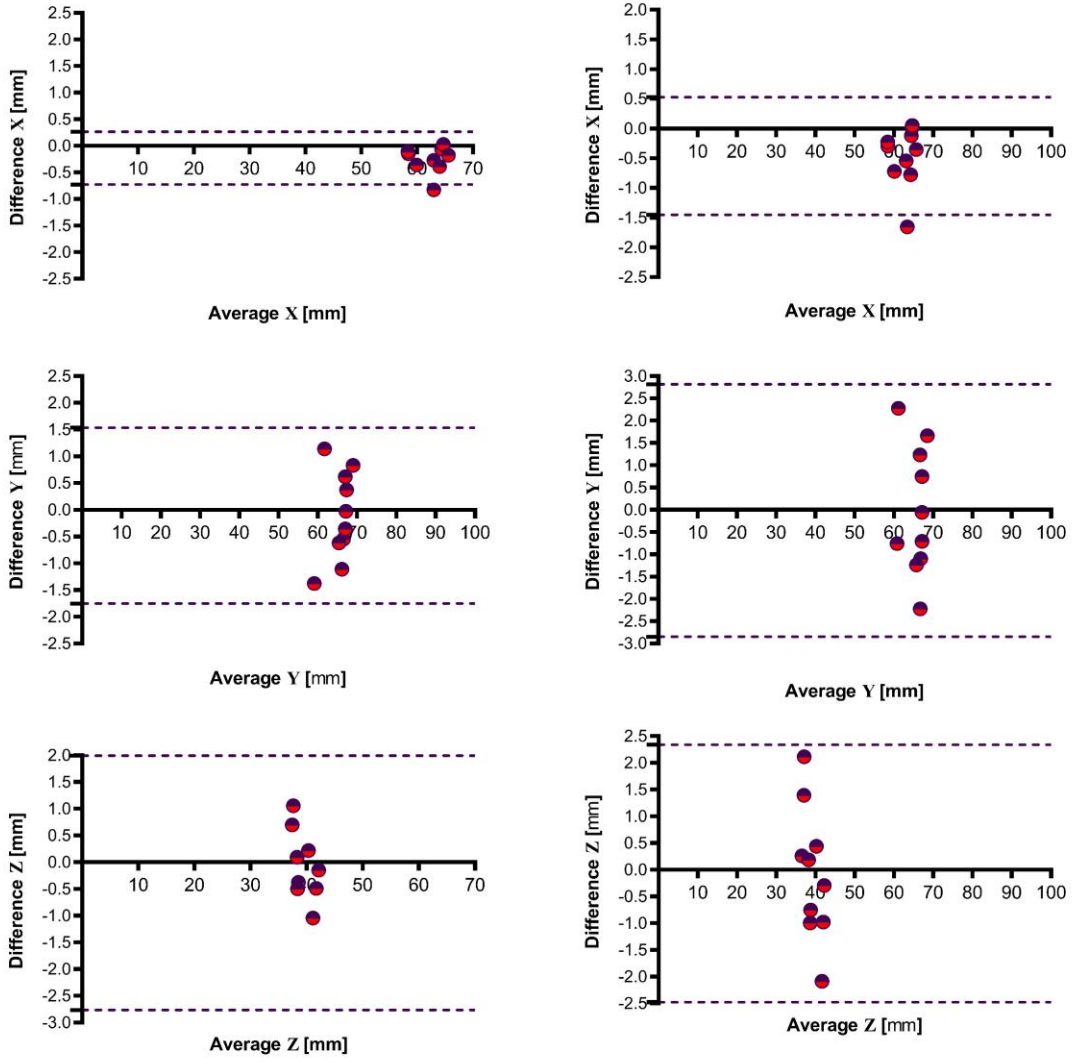

Bland Altman Plot for "CWa Point"

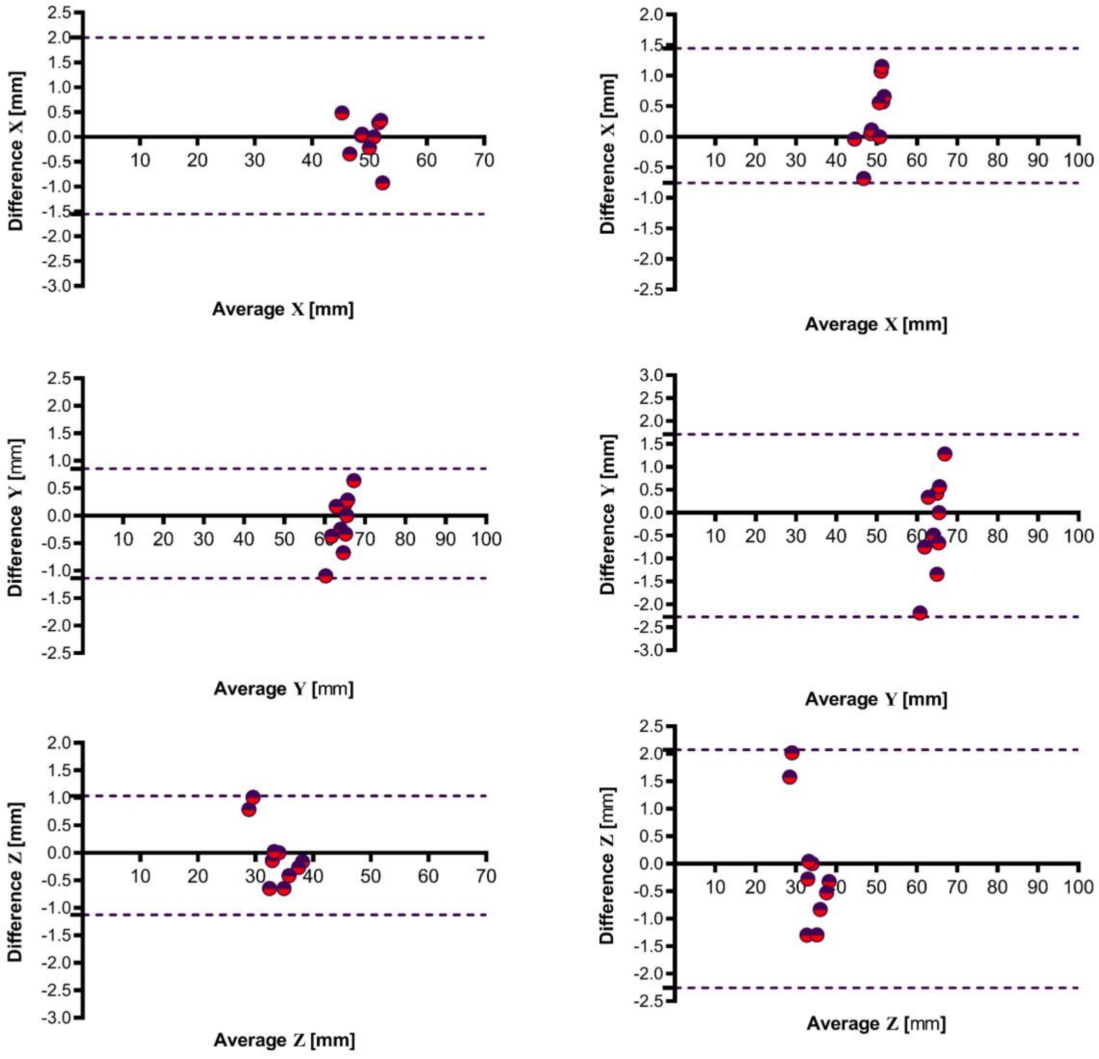

Bland Altman Plot for "CWp Point"

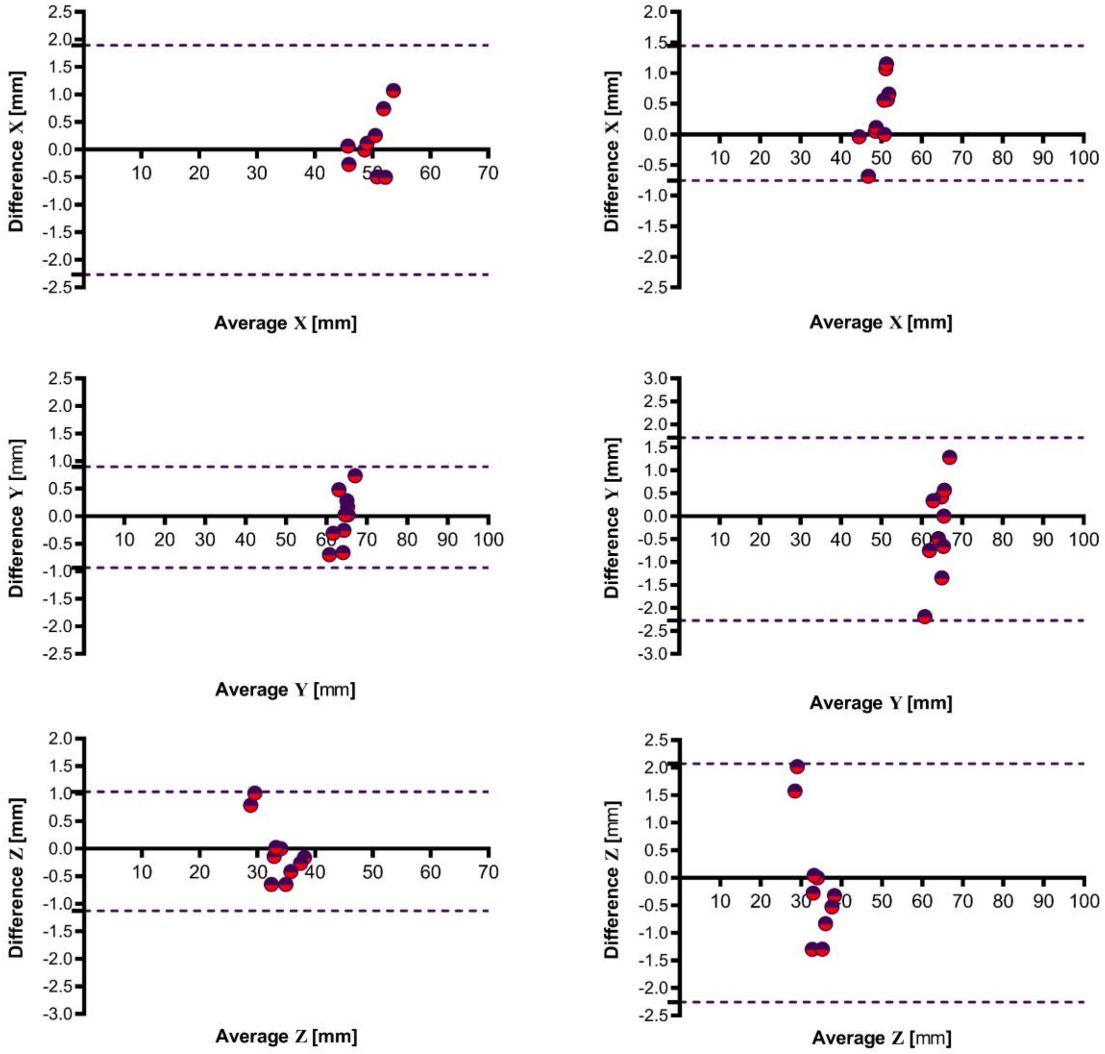

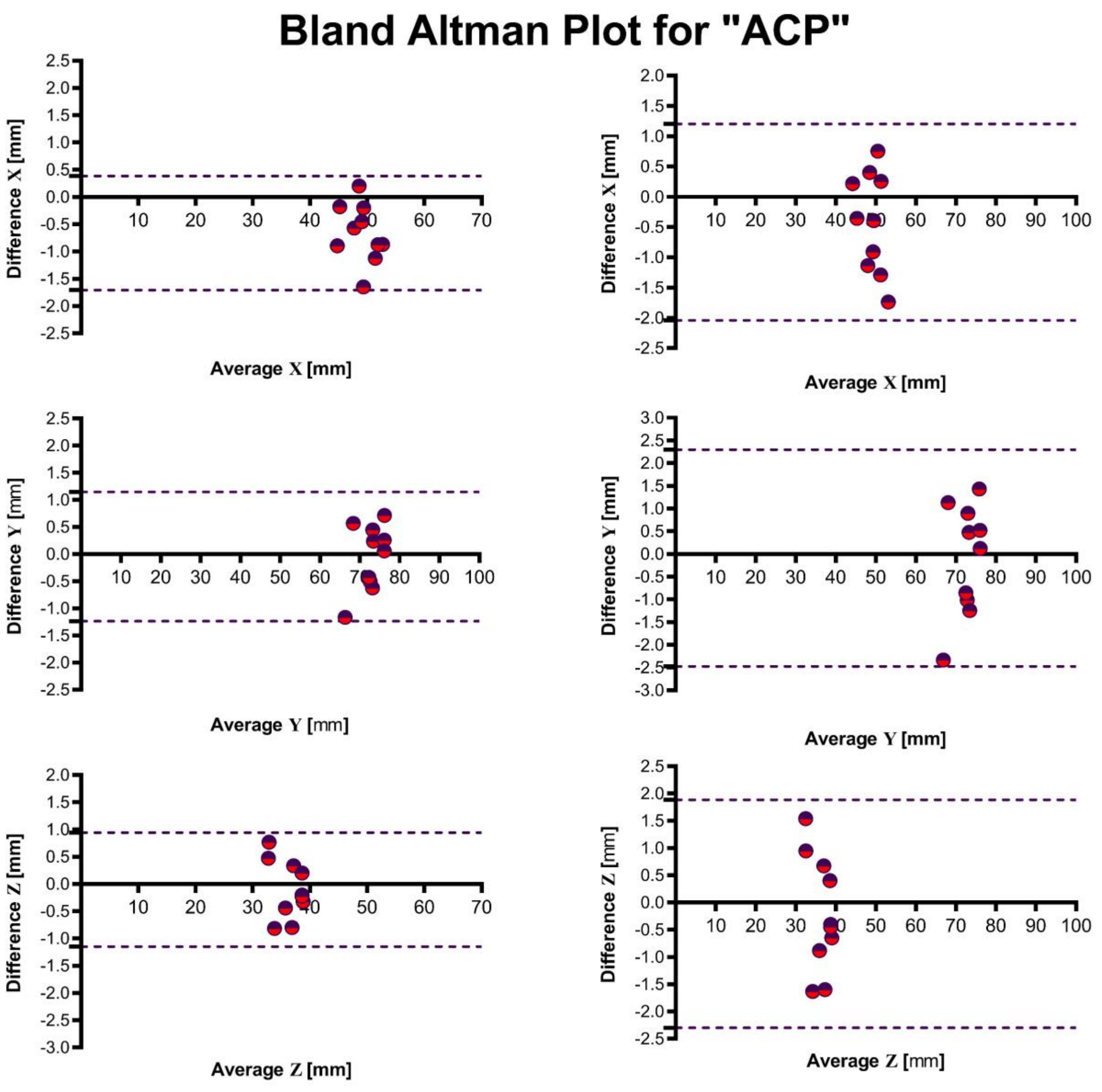
Bland Altman Plot for "ACP"
Difference X [mm]
Average X [mm]
Difference Y [mm]
Average Y [mm]
Difference Z [mm]
Average Z [mm]

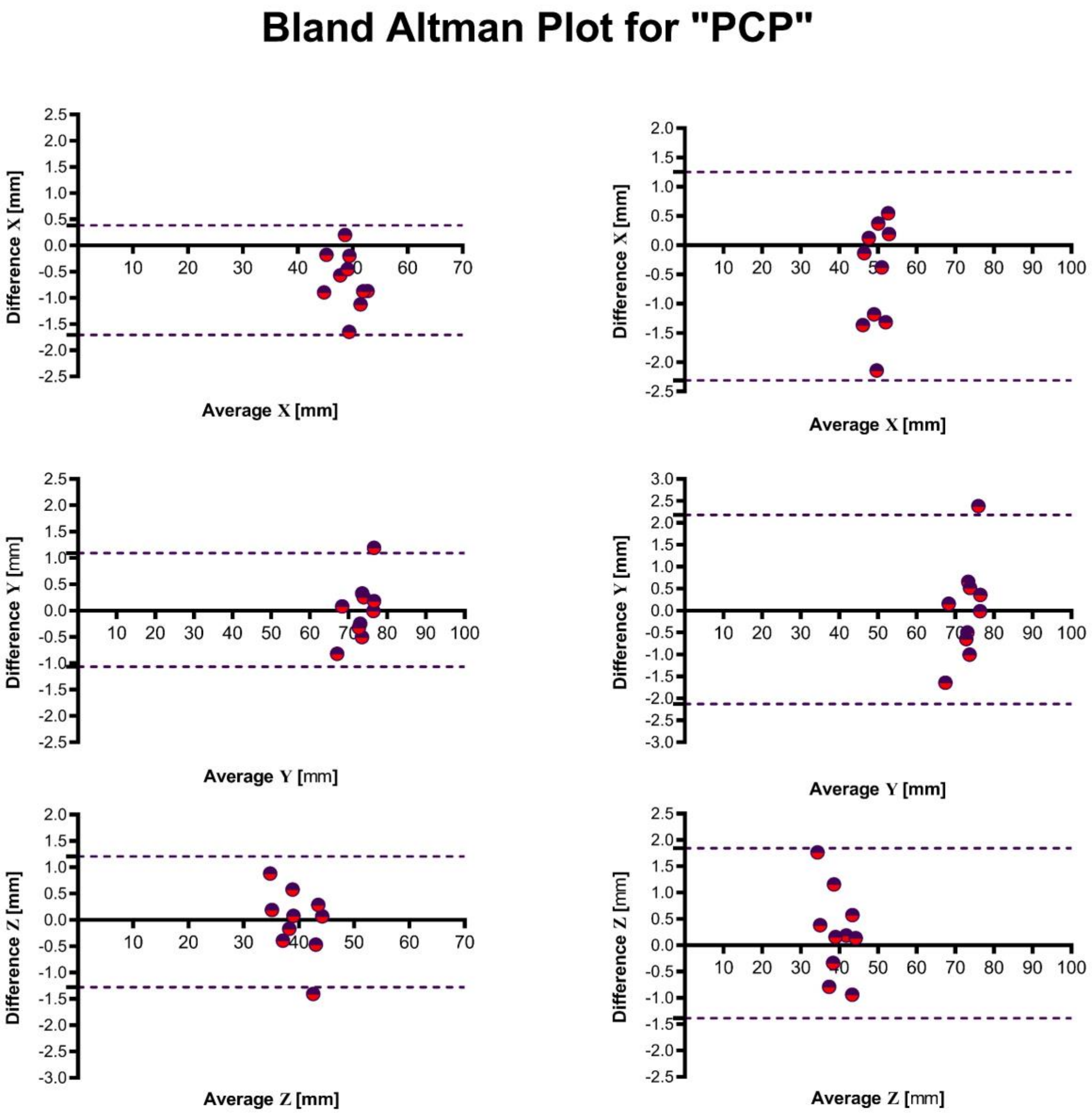
Bland Altman Plot for "PCP"
Difference X [mm]
Average X [mm]
Difference Y [mm]
Average Y [mm]
Difference Z [mm]
Average Z [mm]

Bland Altman Plot for "AT Point"

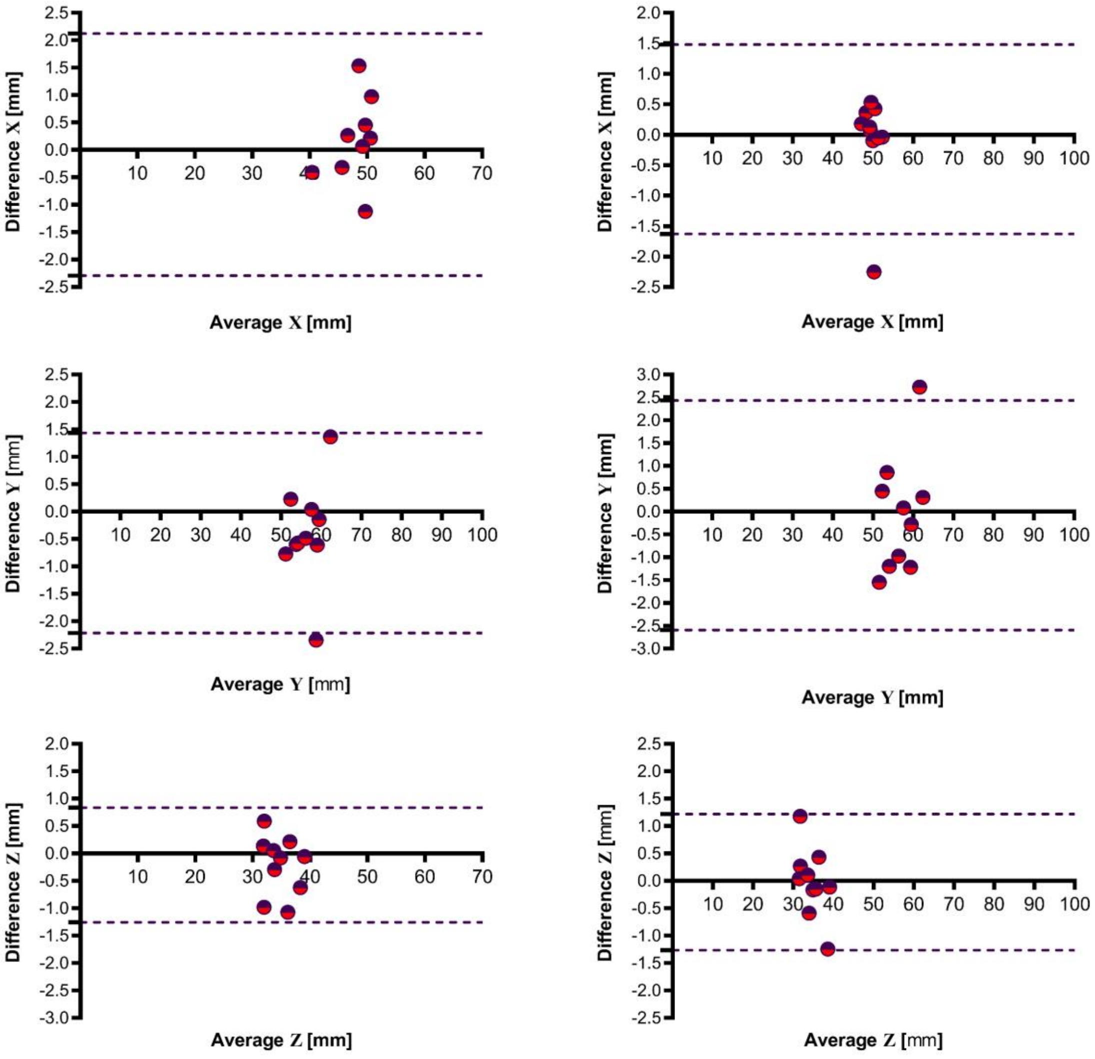

Bland Altman Plot for "IM Point"

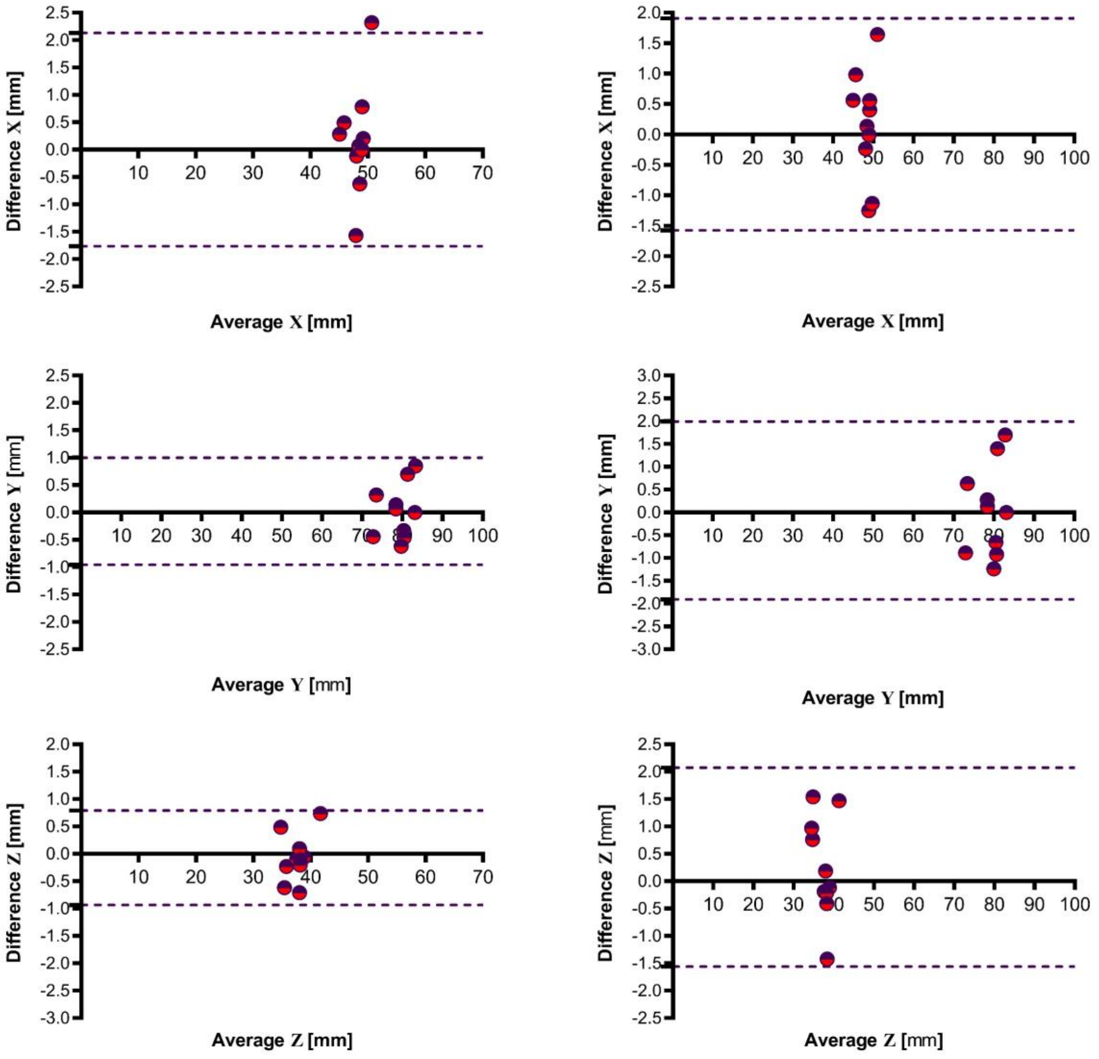

Bland Altman Plot for "AF Point"

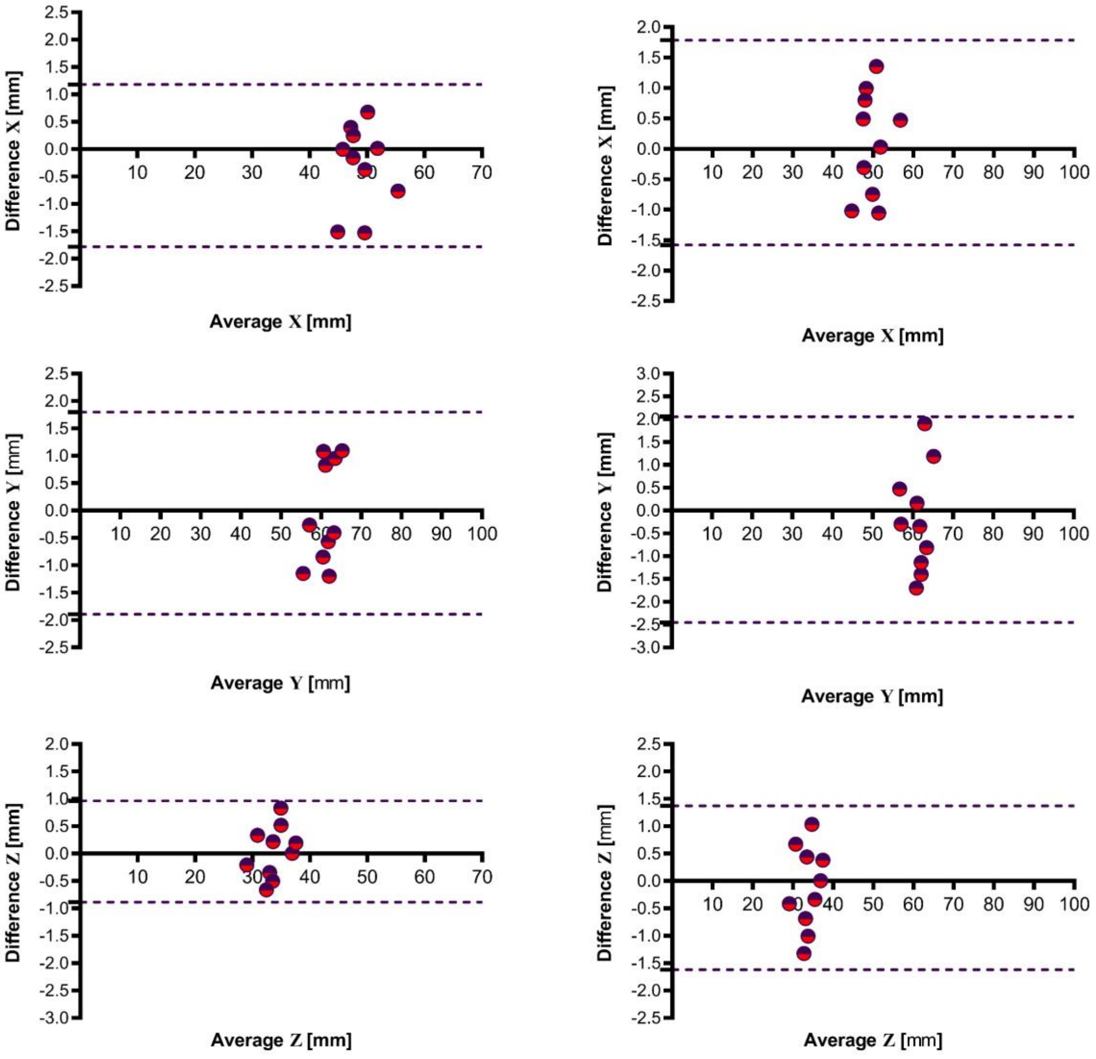

Bland Altman Plot for "PF Point"

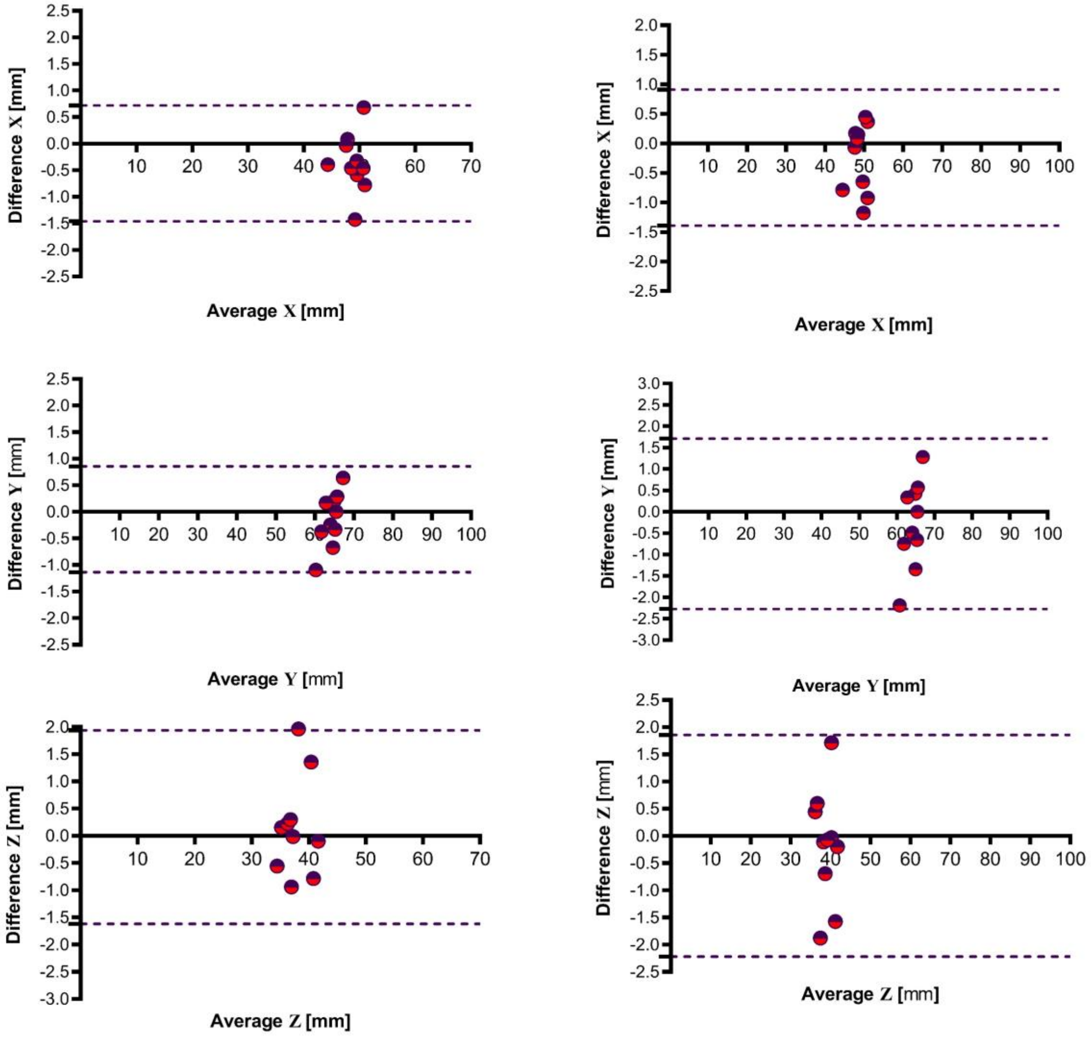

Bland Altman Plot for "ANP Point"

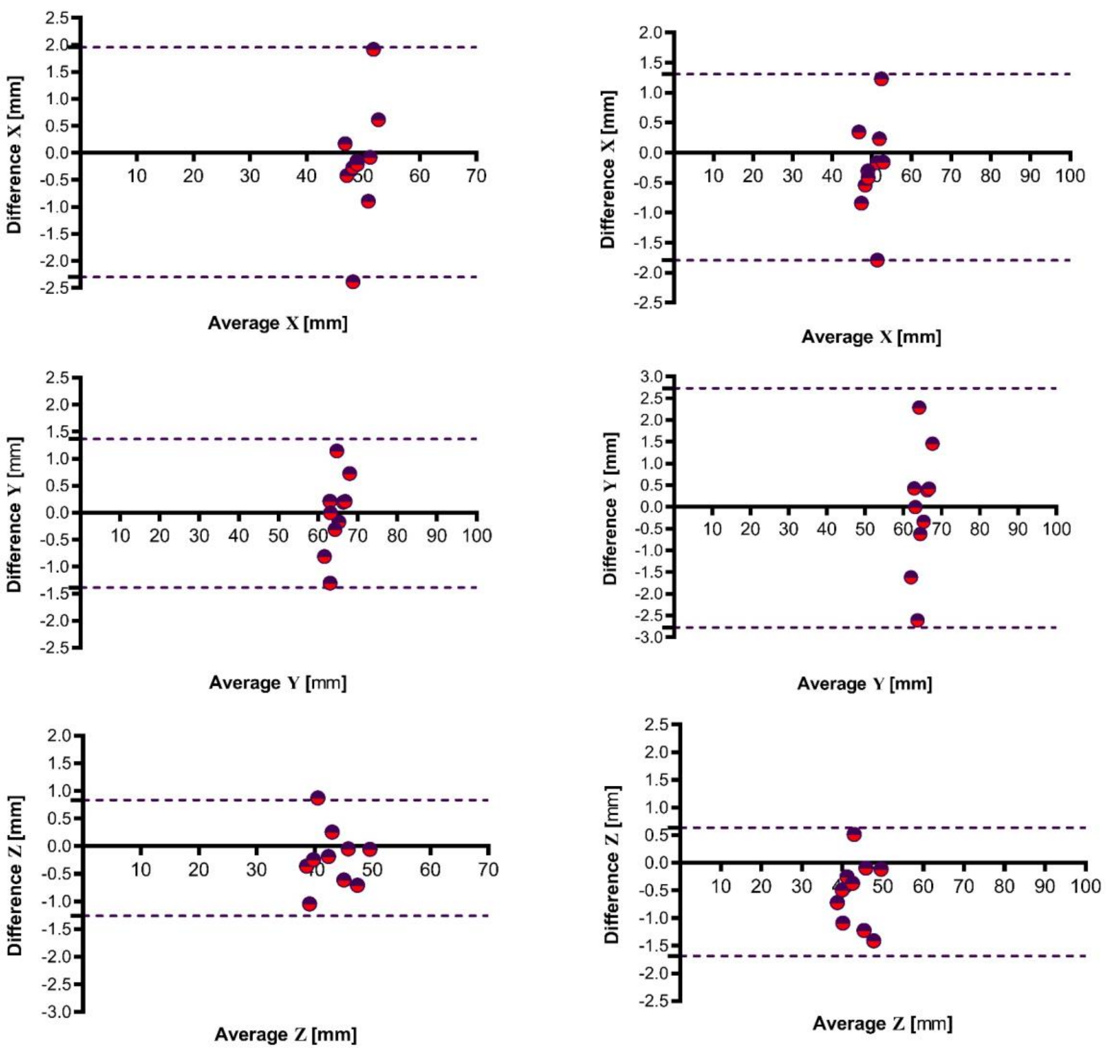

Bland Altman Plot for "PNP Point"

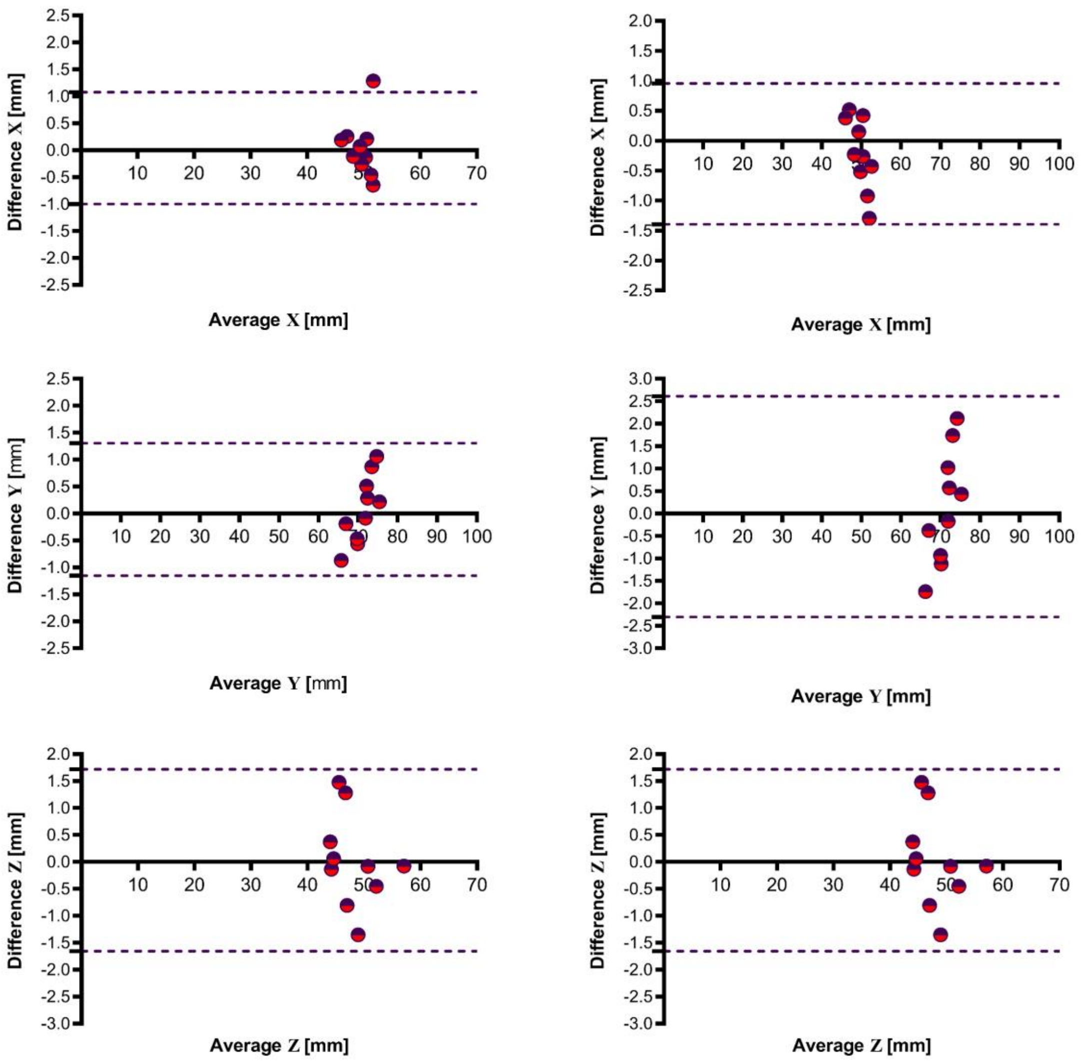

Bland Altman Plot for "AJSf Point"

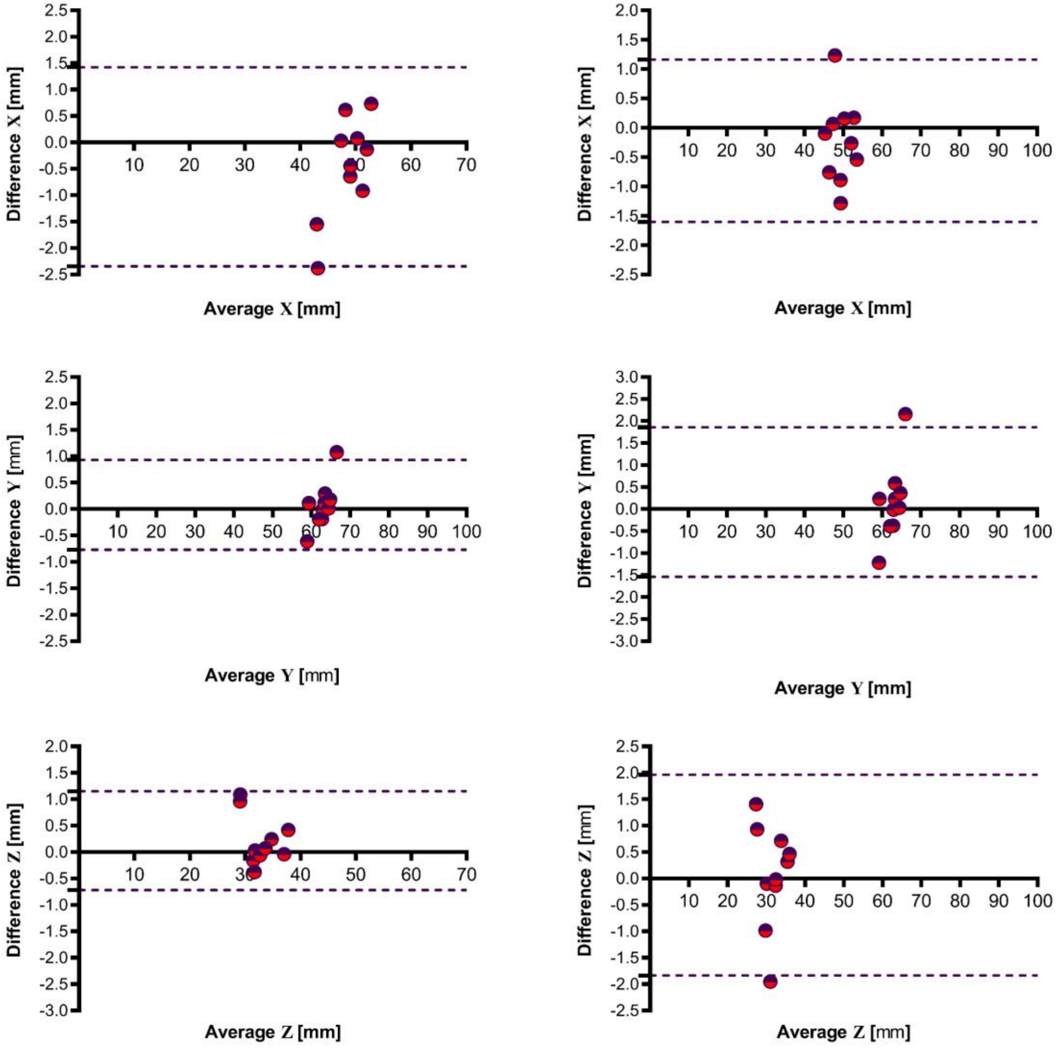

Bland Altman Plot for "AJSc Point"

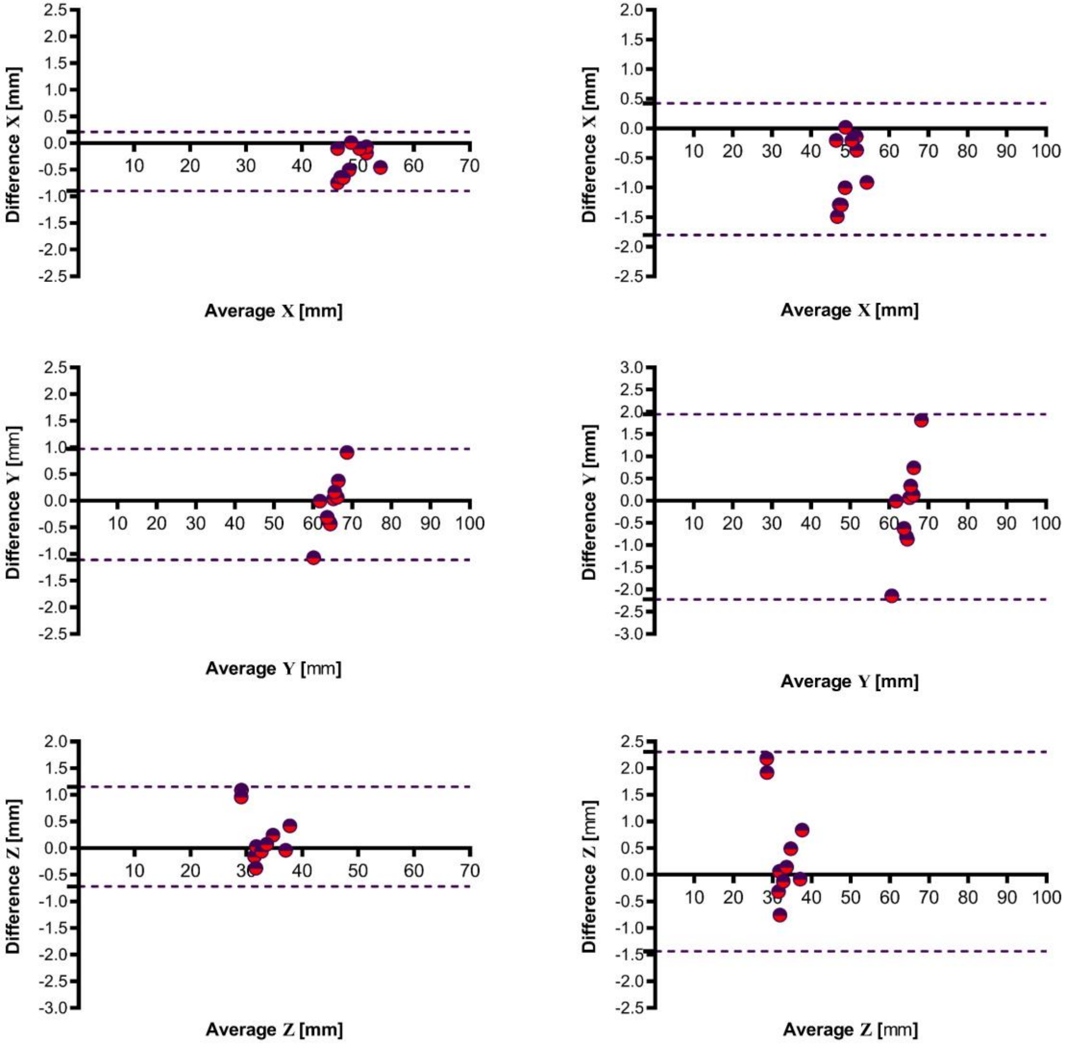

Bland Altman Plot for "PJSf Point"

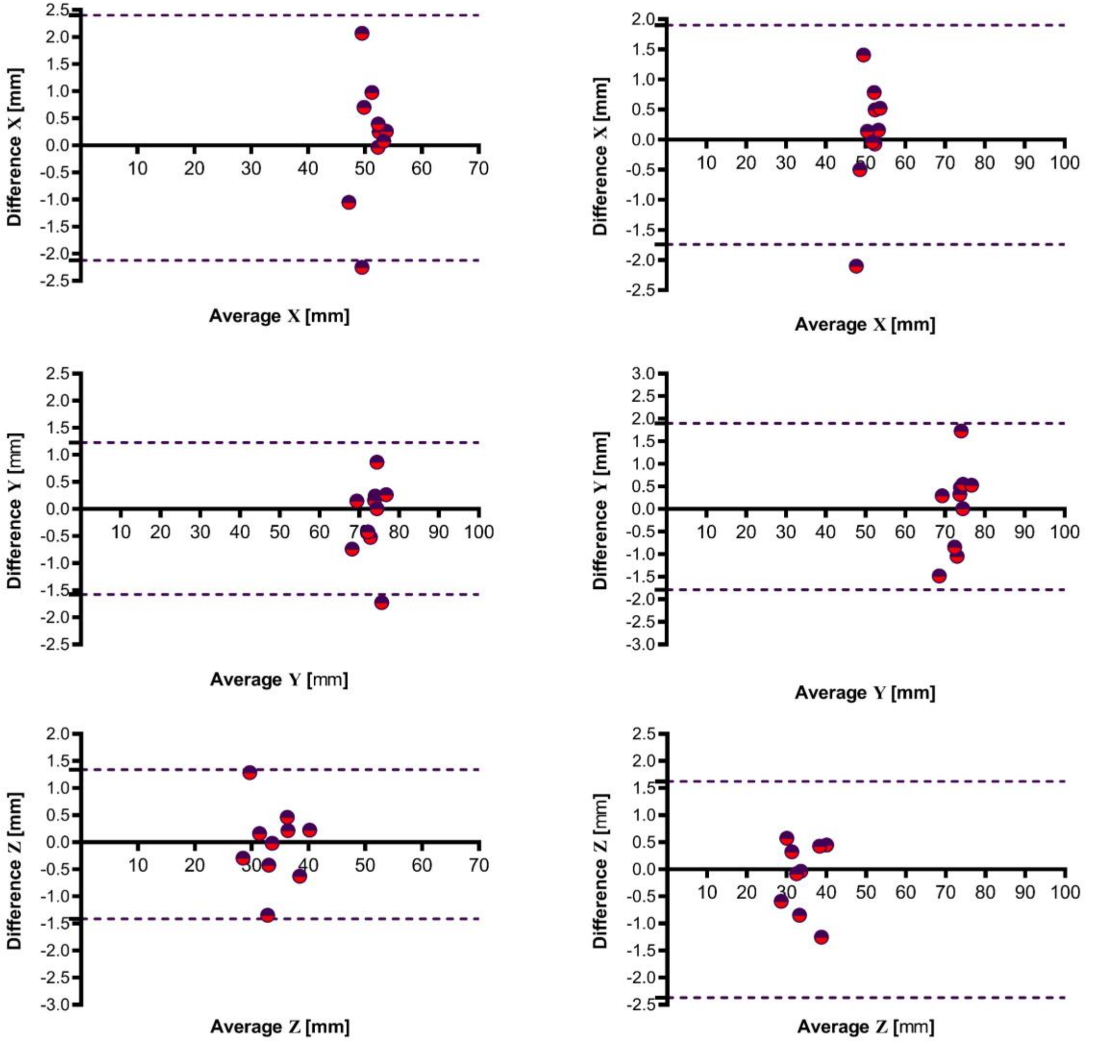

Bland Altman Plot for "PJSc Point"

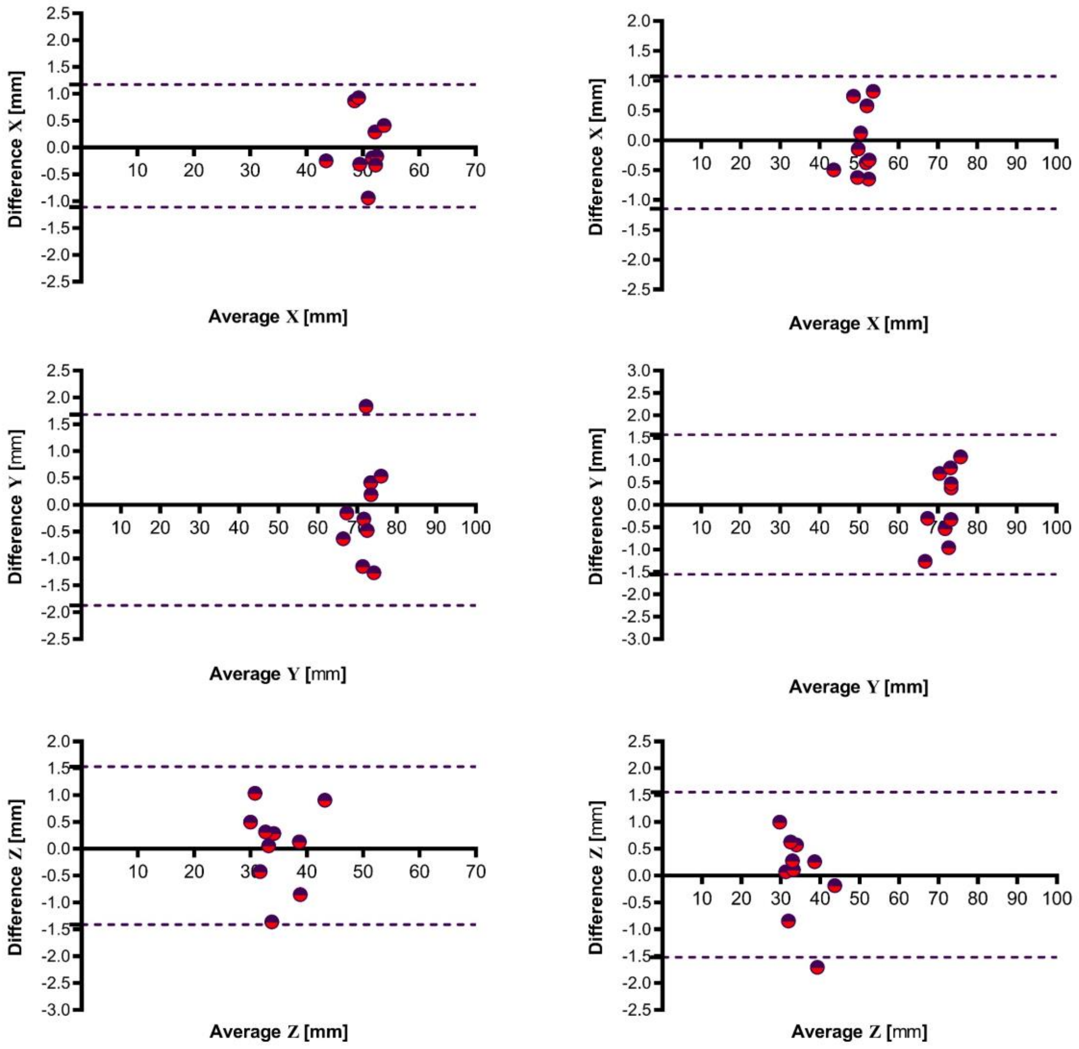

Supplement: Supplementary file 1 — Additional file 1. Appendix A: Supplementary data. [file 40510_2023_474_MOESM1_ESM.pdf]
